# Silver–bismuth perovskite-inspired materials: chemistry, optoelectronic properties, and emerging applications in photovoltaics and beyond

**DOI:** 10.1039/d5ta06180f

**Published:** 2025-10-20

**Authors:** G. Krishnamurthy Grandhi, Noolu. Srinivasa Manikanta Viswanath, Marcello Righetto, Sara Domenici, Mokurala Krishnaiah, Marco Moroni, Adriana Pecoraro, Ana Belén Muñoz-García, Michele Pavone, Lorenzo Malavasi, Teresa Gatti, Paola Vivo

**Affiliations:** a Hybrid Solar Cells, Faculty of Engineering and Natural Sciences, Tampere University P.O. Box 541 Tampere FI-33014 Finland paola.vivo@tuni.fi; b Division of Materials Science and Engineering, Hanyang University 222 Wangsimni-ro Seongdong-gu Seoul 04763 Republic of Korea Hybrid; c Department of Chemical Science, University of Padova Via Marzolo 1 I-35131 Padova Italy; d Department of Applied Science and Technology, Politecnico di Torino Corso Duca degli Abruzzi 24 10129 Torino Italy; e Department of Chemistry and INSTM, University of Pavia Via Taramelli 12 27100 Italy; f Department of Physics “Ettore Pancini”, University of Naples Federico IIComp. Univ. Monte Sant'Angelo Naples 80126 Italy; g Department of Chemical Sciences, University of Naples Federico IIComp. Univ. Monte Sant'Angelo Naples 80126 Italy; h Center for Materials Research, Justus Liebig University Heinrich-Buff-Ring 17 35392 Giessen Germany

## Abstract

Silver–bismuth perovskite-inspired materials (Ag–Bi PIMs) encompass halide double perovskites, vacancy-ordered Cs_2_AgBi_2_I_9_, the (Cu)–Ag–Bi–I family, and structurally related chalcogenides and mixed-anion chalcohalides. Despite their structural diversity, these materials share key electronic features with lead halide perovskites, such as octahedral MX_6_ motifs and similar band edge physics, and have emerged as promising non-toxic alternatives. This review explores the structural and chemical diversity of this semiconductor family, showing how cation disorder (CD), crystal vacancies, and reduced electronic dimensionality (ED)—leading to flat bands and heavy carriers—contribute to their indirect bandgaps, high exciton binding energies, and moderate charge-carrier mobilities. Recent advances in defect passivation, CD engineering, and ED control have led to promising photovoltaic efficiencies (∼10% for AgBiS_2_ under 1 sun illumination and ∼8% for Cs_2_AgBi_2_I_9_ under indoor lighting), alongside unique functional properties, such as pronounced second-harmonic generation, broadband photocatalysis, resistive switching for neuromorphic devices, and high-sensitivity X-ray detection. Emerging insights reveal that homogeneous CD can reduce bandgaps and enhance light absorption, while controlled crystal vacancies induce local structural modifications critical for nonlinear optical responses. By systematically linking the atomic-scale structure to photophysical behaviour and device-level performance, this review traces clear design guidelines—such as enhancing the ED, minimizing deep trap states, and leveraging mixed-anion chemistry—to advance Ag–Bi PIMs from promising lead-free absorbers to versatile platforms for sustainable energy, photonics, and intelligent electronics. We propose a roadmap outlining a three-stage development model focused on material innovation and device optimization for system-level integration, positioning Ag–Bi PIMs as environmentally friendly semiconductors with broad potential in next-generation optoelectronics.

## Abstract 2

We review Ag–Bi perovskite-inspired materials, linking structures, defects, processing, and stability to performance and applications like photovoltaics, X-ray detectors, memory devices, and nonlinear optics.

## Graphical abstract

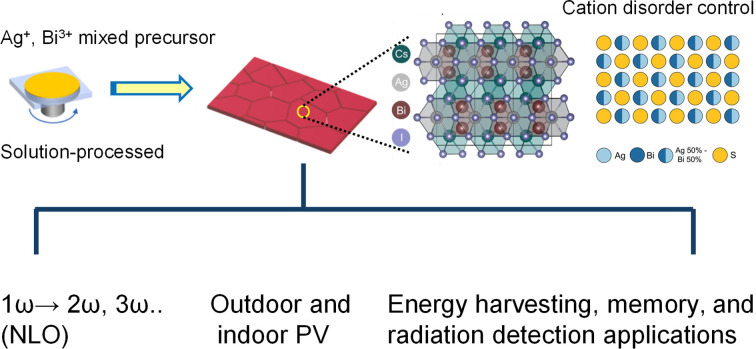

## 1. Introduction

Lead halide perovskite (LHP) solar cells have advanced rapidly over the past decade, reaching record power conversion efficiencies (PCEs) exceeding 26%.^1^ However, the reliance on toxic and water-soluble lead salts has raised significant environmental and regulatory concerns,^2^ driving intense research into stable, environmentally benign alternatives. Lead-free strategies—such as substituting Pb^2+^ with the isovalent cation Sn^2+^—has proved chemically unstable, as Sn^2+^ rapidly oxidizes to Sn^4+^, leading to structural degradation. In contrast, trivalent bismuth (Bi^3+^) has emerged as a compelling alternative due to its non-toxicity, robust air stability, and critical 6s^2^ valence electron configuration, responsible for the defect-tolerant electronic structures central to the superior performance of Pb-based perovskites.^3^ This unique ns^2^ chemistry positions Bi-based perovskite-inspired materials (PIMs) as promising candidates for delivering efficient, stable optoelectronic device performance.^4^

A particularly effective strategy to harness the potential of Bi^3+^ involves pairing it with monovalent silver cations (Ag^+^), forming a diverse family of Ag–Bi PIMs. This heterovalent Ag^+^–Bi^3+^ combination maintains charge neutrality, as the combination of Ag^+^ and Bi^3+^ approximates that of 2 Pb^2+^ ions, enabling stable halide-based crystal structures such as double perovskites (elpasolite-type, *e.g.*, Cs_2_AgBiBr_6_), layered variants (*e.g.*, Cu_2_AgBiI_6_, also known as CABI), vacancy-ordered perovskite derivatives (*e.g.*, Cs_2_AgBi_2_I_9_), and rudorffite-type phases.^5–8^ Ag–Bi PIMs are further categorized into three distinct chemical families based on their anion chemistry—halides, chalcogenides, and chalcohalides. Halide-based compounds such as Cs_2_AgBiBr_6_ possess wide indirect bandgaps (∼2.0–2.2 eV) alongside remarkable air stability.^7^ In contrast, chalcogenides (*e.g.*, AgBiS_2_) feature narrower direct bandgaps (∼1.0–1.2 eV), exceptionally strong optical absorption, and intrinsic chemical stability, making them highly attractive for solar cell applications.^9^ Chalcohalides, combining halide and chalcogenide anions (*e.g.*, AgBiSCl_2_ or sulfur-alloyed halides), bridge these extremes, enabling tuneable optoelectronic properties and optimized bandgaps (∼1.6–2.0 eV).^10^ By systematically studying these distinct chemistries, researchers are unveiling how compositional variation influences electronic dimensionality (ED), defect chemistry, and cation ordering—key factors that govern carrier transport and device performance.

Despite their promising attributes, unlocking the full potential of Ag–Bi PIMs requires overcoming fundamental scientific challenges that limit their optoelectronic functionality. Most notably, the combination of filled d^10^ (Ag^+^) and s^2^ (Bi^3+^) orbitals frequently leads to weak orbital connectivity, creating electronically isolated sub-lattices and low ED. This structural-electronic scenario manifests through indirect or quasi-indirect bandgaps, high exciton binding energies, and low charge-carrier mobilities. Additionally, intrinsic cation disorder (CD)—arising from the similar ionic radii of Ag^+^ and Bi^3+^—and associated antisite defects and vacancies generate deep-level traps and extensive band-edge tailing, severely limiting carrier lifetimes and device efficiency. Recent developments, however, demonstrate that strategic compositional tuning (*e.g.*, Cu or Sb substitutions), refined film processing, and advanced defect management are effective approaches for significantly improving the electronic connectivity and mitigating these limitations.

Intriguingly, the same structural and electronic properties that challenge traditional photovoltaic performance can be advantageous in emerging technology domains. The wide bandgaps (∼1.8–2.2 eV) and defect-tolerant nature of Ag–Bi halide PIMs enable encouraging indoor photovoltaic (IPV) performance, exemplified by Cs_2_AgBi_2_I_9_, reaching PCEs up to ∼8% under indoor LED illumination.^8^ Similarly, AgBiS_2_ solar cells now demonstrate outdoor efficiencies exceeding 10%, driven by optimized CD engineering and extraordinary absorption coefficients.^11^ Beyond photovoltaics, these materials exhibit fascinating multifunctionality. Disorder-driven local symmetry breaking in Cu–(Ag)–Bi–I crystals yields unusually strong nonlinear optical (NLO) phenomena, such as significant second-harmonic generation (SHG), valuable for frequency conversion and integrated photonics.^12^ Ionic conductivity and resistive-switching behaviours in CABI-based compounds further underpin promising memristor and neuromorphic device applications.^13^ Additionally, heavy atomic constituents and charge-carrier self-trapping processes position Ag–Bi halides as attractive candidates for radiation detection and scintillator technologies, significantly broadening their technological scope.^14,15^

Given the rapid diversification and accelerating research momentum on Ag–Bi PIMs since 2024—including breakthroughs such as record indoor efficiencies approaching 8% (Cs_2_AgBi_2_I_9_), outdoor efficiencies exceeding 10% (AgBiS_2_), exceptional NLO responses in Cu–(Ag)–Bi–I systems, and the memristors based on CABI—an up-to-date and comprehensive review is now essential. Although recent reviews, notably by Zhu *et al.* (2024) on Cu–(Ag)–Bi–I semiconductors^16^ and Grandhi *et al.* (2023) on pnictogen-based halide PIMs,^17^ have broadened the scope beyond photovoltaics, a deeper integration of state-of-the-art insights into ED, CD, and defect chemistry across the full halide–chalcohalide–chalcogenide spectrum remains necessary. This minireview precisely bridges this gap. By systematically correlating fundamental structure–property relationships with representative device demonstrations (*e.g.*, photovoltaics, photocatalysis, memristors, and nonlinear optics), we extract practical material design principles to accelerate the development of Ag–Bi PIMs toward impactful applications in sustainable energy conversion, multifunctional optoelectronics, and emerging electronic and photonic technologies.

## 2. Structural aspects

**Fig. 1:**
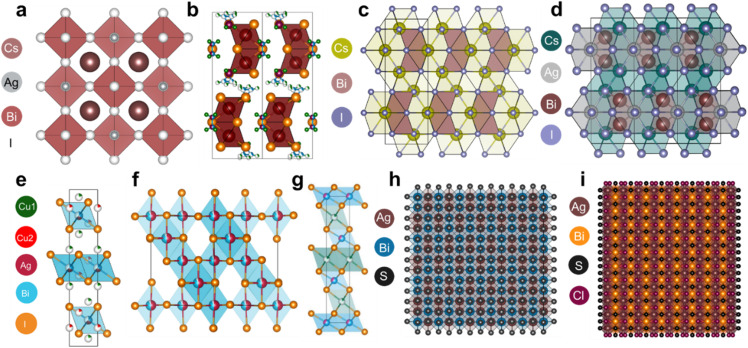
Crystal structures of various Ag–Bi PIMs and related compounds. (a) 3D double perovskite structure of Cs_2_AgBiBr_6_, featuring corner-sharing [AgBr_6_] and [BiBr_6_] octahedra. (b) 2D layered perovskite (BA)_2_AgBiBr_6_, where BA = butylammonium, showing alternating inorganic slabs separated by organic spacer (BA) layers. Reproduced with permission from ref. 22. Copyright 2025, American Chemical Society. (c) Layered vacancy-ordered perovskite Cs_3_Bi_2_I_9_. (d) Layered Cs_2_AgBi_2_I_9_ structure with face-sharing octahedra and a non-centrosymmetric arrangement. (e) Crystal structure of CABI with a mixed Cu/Ag/Bi framework and distorted octahedra. (f) Quasi-1D chain-like structure of AgBiI_4_, highlighting the linear connectivity. (g) Layered Ag_2_BiI_5_ composed of interconnected polyhedra forming 2D planes. The panels (e–g) are reproduced with permission from ref. 12. Copyright 2025. The Author(s). Published by Elsevier Inc. (h) Ordered superstructure of AgBiS_2_, consisting of edge-sharing [AgS_6_] and [BiS_6_] units. (i) Disordered AgBiSCl_2_ lattice with mixed S/Cl anions and CD, leading to structural complexity.

Ag–Bi-based PIMs span diverse structural classes, which are distinguished by symmetry, cation ordering, dimensionality, vacancy distribution, and anion chemistry. In the double perovskite structure, the B-site refers to the central metal cation in the characteristic ABX_3_ framework, where ‘A’ is a monovalent cation (*e.g.*, Cs^+^), ‘B’ is a trivalent or divalent metal (*e.g.*, Ag^+^ and Bi^3+^), and ‘X’ is a halide or chalcogenide anion.^18^ In double perovskites like Cs_2_AgBiBr_6_, two different B-site cations—Ag^+^ and Bi^3+^—occupy alternating positions in a rock-salt pattern, forming a three-dimensional (3D) elpasolite-type lattice.^19^ Their two-dimensional (2D) structural analogues, such as (BA)_2_AgBiBr_6_, incorporate bulky organic cations that separate the Ag–Bi–X layers, reducing both the structural dimensionality and ED. In contrast, vacancy-ordered structures like Cs_2_AgBi_2_I_9_ intentionally leave some B-sites unoccupied to maintain the charge balance and structural stability,^20^ further enriching the structural landscape of Ag–Bi-based PIMs. The (Cu)–Ag–Bi–I family (CABI, AgBiI_4_, and Ag_2_BiI_5_) adopts layered octahedral frameworks. Ag–Bi chalcohalides and chalcogenides (AgBiSCl_2_ and AgBiS_2_) use mixed or pure chalcogenide frameworks, altering their electronic and structural behaviour. These structural features critically influence defect tolerance and charge transport, shaping the optoelectronic potential of Ag–Bi PIMs. The well-known halide elpasolite Cs_2_AgBiBr_6_ is a cubic Ag–Bi double perovskite (*Fm*3̄*m*) with rock-salt ordering of Ag^+^ and Bi^3+^ (Fig. 1a), which lowers the symmetry and shapes its electronic structure.^21^ Its 2D analogue, (BA)_2_AgBiBr_6_ (monoclinic *P*2_1_/*c*), retains cation ordering within perovskite slabs separated by bulky BA^+^ cations (Fig. 1b).^22^ This layering breaks 3D connectivity, introduces octahedral tilting, and enlarges the indirect bandgap due to increased VBM-CBM mismatch. The result is a high exciton binding energy and strong charge-carrier localization. Although moisture-stable, its wide bandgap of ∼2.3 to 2.5 eV and poor interlayer transport hinder its application in photovoltaics. Another important class is the vacancy-ordered double perovskite Cs_3_Bi_2_I_9_, which can crystallize in either a structurally zero-dimensional (0D) dimer phase (*P*6_3_/*mmc*) or a 2D layered phase (*C*2/*c*), depending on the synthesis conditions. Here, half of the B-sites are vacant, leading to isolated or layered [Bi_2_I_9_]^3−^ clusters separated by Cs^+^ ions (Fig. 1c). The lack of 3D octahedral connectivity severely restricts charge-carrier transport, resulting in a large bandgap and low mobility of 2.4–2.5 eV and ∼10^−2^ to 10^−1^ cm^2^ V^−1^ s^−1^, respectively.^23–25^ However, partial substitution of Cs^+^ with Ag^+^, forming Cs_2_AgBi_2_I_9_-type compositions, significantly improves the electronic properties. Ag^+^ incorporation introduces rock-salt-type ordering between Ag^+^ and Bi^3+^ on B-sites, reinstating 3D connectivity and enhancing structural dimensionality (Fig. 1d). Moreover, Ag^+^ alloying reduces the formation of deep trap states associated with isolated [Bi_2_I_9_]^3−^ units by improving lattice continuity, making Ag–Bi hybrid phases superior optoelectronic materials compared to their vacancy-ordered parent.

The (Cu)–Ag–Bi–I family (*e.g.*, CABI, AgBiI_4_, and Ag_2_BiI_5_) shows complex structures shaped by CD and vacancies, which significantly impact their optoelectronic properties. CABI (*R*3̄*m*) features layered, more precisely classified as a rudorffite-type phase characterized by a CdCl_2_-like octahedral motif, defect-perovskite-like structures with Cu^+^, Ag^+^, and Bi^3+^ sharing B-sites, leading to significant CD and vacancies (Fig. 1e).^12^ These induce potential fluctuations, broaden band edges, and create trap states that limit mobility, although the material retains a wide bandgap (∼2.0 eV). AgBiI_4_ (*Fd*3̄*m*) has a cubic rock-salt structure with half of the B-sites being vacant and Ag/Bi sharing sites (Fig. 1f),^12^ but antisite defects and vacancy disorder introduce deep traps, reducing the carrier lifetime and open-circuit voltage (*V*_OC_). Although Ag_2_BiI_5_ and NaVO_2_ share the *R*3̄*m* space group, their octahedral motifs differ: NaVO_2_ features edge-sharing VO_6_ octahedra, while Ag_2_BiI_5_ adopts a rudorffite-type structure with CdCl_2_-like edge-sharing [BiI_6_] and [AgI_6_] units. This highlights that identical symmetry can mask differences in local connectivity—Ag_2_BiI_5_ has higher Ag content and more disorder (Fig. 1g),^12^ promoting non-radiative recombination and poor diffusion lengths despite a narrower bandgap. Among these, CABI stands out for its favourable bandgap, good absorption, and potential for defect engineering, making it more promising for photovoltaic applications despite some structural disorder.

Chalcogenide PIMs such as AgBiS_2_ and AgBiS_*x*_Se_1−*x*_ are another important Ag–Bi material family. The structural and electronic properties of AgBiS_2_ are strongly influenced by its intrinsic CD and the formation of sulfur vacancies (V_S_).^26^ AgBiS_2_ typically crystallizes in a rock-salt-derived structure with cubic symmetry, most commonly associated with the *Fm*3̄*m* space group (No. 225), akin to the ideal NaCl-type lattice. While the fully disordered schapbachite phase is stable at room temperature due to entropy-driven CD, the ordered matildite phase with checkerboard A-site occupation is also stable, stabilized by favourable electrostatic interactions between Ag^+^ and Bi^3+^. In AgBiS_2_, Ag^+^ and Bi^3+^ statistically share the 4a site, while S^2−^ occupies the 4b site, forming a close-packed lattice (Fig. 1h). The similar ionic radii and moderate charge difference favor CD over long-range ordering. This disorder induces local charge imbalances, particularly in Bi-rich regions, which in turn stabilize sulfur vacancies. These vacancies distort the valence band, introducing deep trap states that facilitate non-radiative recombination, shorten carrier lifetimes, and ultimately limit the photovoltaic efficiency.^27,28^ In contrast to disordered AgBiS_2_, AgBiSCl_2_ crystallizes in an orthorhombic *Cmcm* structure with distinct, ordered Ag^+^ and Bi^3+^ sites. Its layered anion sublattice, with S^2−^ and Cl^−^ ions, favours Bi–S and Ag–Cl coordination (Fig. 1i), promoting both cation and anion ordering. This structural order suppresses the formation of deep-level defects, particularly sulfur vacancies, reducing non-radiative recombination. The anisotropic bonding and orthorhombic distortions enhance lattice stability, exciton binding, and defect tolerance.^10^ Additionally, the mixed-anion framework allows tuneable bandgaps *via* S/Cl electronegativity, making AgBiSCl_2_ a more stable and efficient alternative to AgBiS_2_ for optoelectronic applications.

From a structural perspective, AgBiS_2_ stands out as a highly promising photovoltaic candidate for sunlight harvesting, owing to its optimal direct bandgap (∼1.5 eV), strong absorption, and relatively defect-tolerant chemistry—provided that CD is well-controlled and sulfur vacancy formation is suppressed. CABI is a strong secondary option, though its layered, disordered structure introduces mid-gap states that limit performance without defect management. AgBiSCl_2_ benefits from structural ordering and improved stability but still needs optimization to enhance the carrier mobility. Cs_2_AgBi_2_I_9_ offers better connectivity than Bi-only phases but is hindered by partial vacancy disorder. Other materials like Cs_2_AgBiBr_6_, (BA)_2_AgBiBr_6_, AgBiI_4_, and Ag_2_BiI_5_ are structurally disadvantaged due to large indirect bandgaps, poor transport, and deep trap states, making them less suitable for light-harvesting applications.

### 2.1. Stability of Ag–Bi PIMs

The study on the structural stability of Ag–Bi based PIMs under various environmental conditions has become more important toward their practical application in high-performance and reliable optoelectronic devices because the device performance depends largely on how absorber layers preserve their functionalities under humidity, upon thermal cycling, or owing to degradation related to defects. Whereas previous discussions on stability primarily centered around issues such as Sn^2+^ oxidation in related perovskite systems,^29^ recent efforts have highlighted Ag–Bi PIMs, which demonstrate high stability under relevant operating conditions. For example, Cs_2_AgBiBr_6_ has emerged as a key model material for applications because of its structural stability even when exposed to moisture over an extended period, and hydrophobic surface coatings have been demonstrated to enhance this stability by retardation of hydrolytic processes and suppression in the generation of defects. In addition, thermal stability studies have demonstrated that Cs_2_AgBiBr_6_ and similar compounds maintain their crystalline structure upon numerous recrystallization treatment cycles with associated reversible phase transition without detriment to the optoelectronic properties due to its robust bonding environment and favourable lattice symmetry. In addition to these inherent features, one of the most efficient pathways to suppress recombination channels and improve environmental robustness for long device lifetimes has been realized *via* the defect engineering approaches by deliberately introducing vacancies, ion substitution as well as compositional tunings.^30–32^ Beyond Cs_2_AgBiBr_6_, other homologues have also manifested compelling stability performance: CABI has displayed remarkable resilience against thermal and moisture stresses, enduring them for more than 240 days under ambient conditions;^13,33^ the rudorffite-type iodides AgBiI_4_ and Ag_2_BiI_5_ exhibit tuneable dimensionality and high thermodynamic stability,^34^ paving a way for defect-tolerant device architecture; the scalability of AgBiS_2_ to solution-processed thin films with high photovoltaic performance as well as noteworthy ambient stability is unprecedented among open electron conduction, providing guidance to commercial photovoltaic processes.^35–37^ The non-toxic chalcogenide-halide system of AgBiSCl_2_ confers dual advantages in terms of environmental friendliness and promising operative integrity.^10,38^ Collectively, these results demonstrate that Ag–Bi PIMs integrate native lattice stability and extrinsic approaches (*e.g.*, surface passivation and defect engineering) to realize the environmental tolerance which is competitive with conventional lead-based perovskites. Nevertheless, in contrast to these promising prospects, the underlying mechanisms that trigger their long-term structural degradation are not entirely clear, and there is a great need for systematic studies that link advanced characterization with computational modelling in order to gain insight into defect dynamics, deformations as well as phase stability under realistic device operating conditions. It is only *via* such integrated strategies that the full potential of Ag–Bi PIMs can be realized for reliable applications in future solar cell and optoelectronic applications.

To provide a concise overview, the main Ag–Bi compounds discussed in this review are summarized in Table 1. The table organizes structural families, representative compositions, bandgaps, electronic-dimensionality notes, defect trends, mobility ranges, and benchmark device metrics. This comparative picture complements the structural discussion above and serves as a reference point for the optoelectronic properties, defect chemistry, and device performance that will be discussed in later sections.

**Table 1:** Ag–Bi materials: structural classes and key properties

Ag–Bi family	Representative composition(s)	Structure (space group)	Bandgap (eV) & character	Effective masses 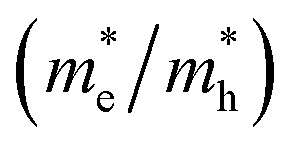	Mobility range (cm^2^ V^−1^ s^−1^)	ED (qualitative)	Dominant native defects	Best device metric (context)
Halide, double perovskite	Cs_2_AgBiBr_6_	Elpasolite (*Fm*3̄*m*)	≈2.2 (indirect); first strong direct transition at higher E	0.33/0.35	∼3 → 1.3 (deloc → loc)	Low ED: VBM (Ag-4d/Br-p) *vs.* CBM (Bi-6p) separation	V_Br_; Ag_Bi_/Bi_Ag_ antisites	6.37% (1-sun, H-treated); ∼7% (1000 lux WLED, IPV)
Vacancy-ordered iodide	Cs_2_AgBi_2_I_9_	Layered, face-sharing motifs	≈1.8 (quasi-direct)	0.6/2.1	—(Mobility-lifetime product (*µ*_*τ*_) of 3.4 × 10^−3^ cm^2^ V^−1^)	Higher ED (quasi-2D) than Cs_3_Bi_2_I_9_*via* Ag–I–Bi connectivity	V_I_ (iodine vacancies)	∼3% (1-sun); ∼8% (1000 lux, IPV)
Cu–Ag–Bi–I	CABI (Cu_2_AgBiI_6_)	Layered CdCl_2_-like (*R*3̄*m*)	≈1.9–2.0 (direct)	0.6/1.0	∼2.1 → 1.0 (deloc → loc)	Cu 3d raises the VBM → improved ED	V_Cu_/V_Ag_ (shallow), V_Bi_ (deeper)	∼2.2–3% (1-sun); 5.66% (1000 lux, IPV)
Iodide (Ag-rich rudorffite)	Ag_3_BiI_6_	Rudorffite-related (edge-sharing octahedra)	≈1.8–2.0 (direct/quasi-direct)	0.4/0.4	∼0.41 → 0.02 (deloc → loc)	Moderate ED; disorder-broadened edges	Antisites + vacancies	∼4.3% (1-sun); 5.6% with S-alloying
Chalcohalide (mixed anion)	AgBiSCl_2_	Orthorhombic (*Cmcm*)	>2.0 (direct)	—/—	—	Intermediate ED; ordered Ag/Cl–Bi/S layers	V_S_ suppressed *vs.* AgBiS_2_; V_Cl_; mixed-anion (S_Cl_/Cl_S_)	Projected >10% (theory) under 1-sun; no PEC demonstrated yet
Chalcogenide	AgBiS_2_	Rock-salt-derived (*Fm*3̄*m*) (CD-tunable)	≈1.5 (direct when homogeneously disordered)	0.35/0.722 (segregated case)	∼0.43 → 0.11 (segregated) to 2.7 → 2.2 (disordered)	ED increases with homogeneous CD; strong absorption	V_S_, antisites; CD-controlled	10.20% (0.06 cm^2^), 9.53% (1.00 cm^2^), and ∼10% NC-based

## 3. Electronic structure, defect chemistry, and optoelectronic properties

### 3.1. Electronic band structure

As discussed in the previous section, various Ag–Bi perovskite-inspired structures exhibit distinct electronic and optical behaviours. A detailed understanding of their electronic structures is therefore crucial for effectively optimizing these materials for diverse device applications. Since Bi^3+^ and Pb^2+^ share similar valence electron configurations (6s^2^), it is often expected that Bi^3+^-based materials exhibit defect tolerance comparable to LHPs, where intrinsic defects typically do not introduce significant deep-level states within the bandgap.

However, Bi^3+^ ions differ significantly from Pb^2+^ ions in having a smaller ionic radius, a higher oxidation state, and deeper-lying 6s^2^ orbitals, all of which lead to notable differences in the lattice structure. These structural differences consequently alter the electronic properties and defect chemistry of Ag–Bi-based materials.

Cations with higher oxidation states (*e.g.*, Bi^3+^ compared to Pb^2+^) introduce more transition levels within the bandgap, thereby enhancing the likelihood of deep trap formation.^39^ Additionally, unlike LHPs where the metal 6s^2^ orbitals directly contribute to the VBM, the Bi^3+^ 6s^2^ lone-pair orbitals in Ag–Bi PIMs lie approximately 1 eV below the valence-band maximum (VBM) and are poorly coupled to the halide p states.^4,39^ This energetic mismatch leads to a less dispersive valence band and higher effective masses for charge carriers, significantly increasing the tendency to form deep trap states. Specifically, as the energetic separation between the cation and anion orbitals grows, dangling-bond orbitals become strongly localized, pushing defect states deeper into the bandgap. Furthermore, shorter spatial distances between neighbouring dangling orbitals—such as those between Bi atoms around an oxygen vacancy compared to those around an iodine vacancy—strengthen the orbital overlap and deepen the resulting trap states (illustrated by shallow iodine vacancies in BiOI *versus* deep oxygen vacancies in Bi–O materials).^39^ Consequently, careful structural and compositional engineering is essential to achieve Ag–Bi PIMs with high defect tolerance.

Ag–Bi double perovskites, such as Cs_2_AgBiBr_6_, typically exhibit an indirect bandgap. This arises from the differing orbital contributions at the VBM and conduction band minimum (CBM): the VBM is primarily composed of anti-bonding Ag 4d and halide p orbitals, while the CBM is dominated by Bi 6p orbitals (with negligible Ag 5s character).^40^ These states occur at different *k*-points in the Brillouin zone, resulting in reduced band-edge absorption. Additionally, Jahn–Teller-like distortions associated with Ag^+^ broaden the density of states and lower the charge-carrier mobility. Antisite defects and Br vacancies further introduce non-radiative recombination pathways, limiting photovoltaic efficiency despite the material's excellent stability. In contrast, Cs_2_AgBi_2_I_9_ exhibits a different electronic structure due to its vacancy-ordered lattice. Here, Ag^+^ (4d^10^) states contribute more significantly to the VBM, raising the VBM energy and narrowing the bandgap. Moreover, Ag 5s and I 6p orbitals contribute to the CBM in Cs_2_AgBi_2_I_9_, forming an intermediate band that further reduces the bandgap.^20^ This greater orbital overlap leads to increased band dispersion and lower effective masses, resulting in enhanced carrier mobility compared to Cs_2_AgBiBr_6_.^8,41^

Recent studies have focused on mixed-metal iodides such as CABI, which exhibit a direct bandgap of ∼2 eV and strong light absorption. In CABI, both theoretical and experimental studies show that the VBM is primarily formed by Cu 3d states, mixed I 5p orbitals—similar to AgBiI_4_—while the CBM is dominated by Bi 6p and I 5p states.^6,42^ This orbital alignment reduces the mismatch at the band edges and increases the optical absorption efficiency of the material. In contrast, in Cu-free Ag–Bi–I compositions, the VBM is dominated by I-5p states.

Bandgap tunability in Ag–Bi PIMs can be achieved through halide mixing. Ag–Bi iodide PIMs typically exhibit smaller bandgaps (∼1.8 eV), which enhance light absorption and charge transport. In contrast, bromide-rich compositions display wider bandgaps and higher exciton binding energies. Recent density functional theory (DFT) studies have shown that mixing of Br with CABI enables fine-tuning of the bandgap from around 1.9 eV (iodine-rich) to about 2.3 eV (bromide-rich), without introducing problematic mid-gap states.^43^ Similarly, modifying the A-site cations (such as using mixtures of Cs^+^, Rb^+^, and formamidinium) also influences the electronic properties. DFT calculations on similar Sb-halide PIMs indicate that mixed A-site cations improve structural stability and reduce energetic disorder at grain boundaries, indirectly enhancing charge transport.^44^ Similar strategies are expected to be effective in Ag–Bi PIMs as well.

Ag–Bi PIMs frequently show notable CD, which will be discussed in detail in Section 3.4. This disorder sometimes can positively influence the electronic structure of the PIMs. For example, CD can broaden electronic bands and create localized energy states at the band edges. Recent theoretical simulations of CABI showed partial ordering of Cu and Ag at room temperature, and this partial ordering directly affects the electronic structure near the band edges.^43^ This quaternary PIM also frequently contains vacancies, particularly at Cu and Ag sites. These vacancies typically form shallow acceptor levels, which may facilitate p-type conductivity. Importantly, such defects generally do not introduce deep trap states. DFT calculations have confirmed this defect-tolerant behaviour, showing a low density of recombination centres.

Ag–Bi halide PIMs can adopt various structural dimensionalities (atom connectivity in spatial coordinates), including 3D double perovskites, 2D layered structures, and even 0D molecular-like configurations. Dimensionality plays a critical role in determining their electronic properties. Low-dimensional structures typically exhibit high exciton binding energies and reduced carrier mobilities, but they can be advantageous for applications such as nonlinear optics and light emission (*e.g.*, scintillators). For instance, CABI possesses a near-3D crystal structure and improved electronic connectivity compared to ternary Ag–Bi–I systems, enabling relatively isotropic hole transport. Theoretical calculations have predicted hole mobilities exceeding 10^−2^ cm^2^ V^−1^ s^−1^ in ideal single crystals.^6^ However, structural complexities arising from mixed-metal sites still introduce anisotropy in the electronic bands, making control over crystallinity during synthesis essential for optimizing performance.

Further tuning of electronic properties can be achieved through strain engineering or element alloying. For example, partially substituting Bi^3+^ with Sb^3+^ lowers the CBM, improving energy level alignment with electron transport layers and enhancing device performance. Studies on antimony–bismuth and Ag–Bi PIM alloys have shown that Sb^3+^ incorporation reduces structural disorder and sharpens absorption edges, resulting in improved photovoltaic efficiency.^45^ Additionally, applying strain, *via* chemical doping or external pressure, can beneficially modify the electronic structure without compromising defect tolerance.

By replacing halides with chalcogenides, the electronic structure of Ag–Bi PIMs is markedly altered in terms of band dispersion, dimensionality, and defect tolerance. Halide-based Ag–Bi PIMs (*e.g.*, Cs_2_AgBiBr_6_) generally feature indirect bandgaps with flat, dispersal-limited band edges due to the spatial separation of Ag-centred and Bi-centred orbitals. In contrast, Ag–Bi chalcogenides such as AgBiS_2_ and AgBiSe_2_ typically exhibit narrower, more direct bandgaps (on the order of 1.0–1.3 eV) and exceptionally strong optical absorption. The greater covalency of Bi–S/Se bonds can promote stronger orbital overlap at the band edges, but the ED in these chalcogenides typically remains low when Ag and Bi cations are ordered or phase-segregated, yielding quasi-0D electronic structures analogous to the halide double perovskites. This often manifests as large exciton binding energies, pronounced carrier localization (self-trapping), and indirect-like optical behaviour in disordered lattices. Notably, AgBiS_2_ crystallizes in a rock-salt-derived structure where Ag^+^ and Bi^3+^ randomly occupy the cation sublattice, and if this CD is homogeneous, it can increasingly lead to more dispersive bands. Indeed, recent experiments show that engineering a more homogeneously disordered AgBiS_2_ (*i.e.*, avoiding Ag/Bi clustering) results in bandgap narrowing and enhanced absorption.^9^ Alloyed chalcogenides AgBiS_*x*_Se_1−*x*_ allow bandgap tuning across 1.0–1.3 eV (absorption in the 700–1200 nm wavelength regime).^46^

Chalcohalide Ag–Bi PIMs, which combine halide and chalcogenide anions (*e.g.*, AgBiSCl_2_), tend to bridge the above behaviours. Due to the mixed anion chemistry, these materials have intermediate bandgaps (often in the 1.1–1.7 eV range) that are direct in nature and moderately dispersive. For instance, AgBiSCl_2_ adopts an ordered orthorhombic structure with alternating Bi–S and Ag–Cl layers, yielding a direct bandgap at the Brillouin zone *Y*-point and greater in-plane band dispersion than the fully halide analogues. Orbital-projected density of states and charge density analyses reveal that the valence band of AgBiSCl_2_ is predominantly composed of sulfur and chlorine p orbitals, along with significant contributions from silver d orbitals. In contrast, the conduction band is chiefly governed by bismuth 6p orbital character. The incorporation of the more electronegative halide (Cl^−^) alongside S^2−^ also allows tuning of band-edge positions *via* anion electronegativity differences.^10^

Importantly, AgBiSCl_2_ is reported to be a more structurally stable and defect-tolerant semiconductor than pure AgBiS_2_. In AgBiS_2_, sulfur vacancies (V_S_) are the dominant native defects (owing to the relatively weak Ag–S and Bi–S bonds) and can act as deep traps under S-poor conditions.^26^ By contrast, the presence of a halide in AgBiSCl_2_ may suppress or compensate such anion-vacancy formation, leading to fewer mid-gap states and consequently a clear band-edge photoluminescence (PL),^10^ whereas AgBiS_2_ typically exhibits only broadband trap-state dominant emission.^47^ Overall, replacing halides with chalcogenides tends to narrow the bandgap and increase optical absorption but at the cost of more localized carriers and potential deep defect states, while chalcohalide materials offer a compromise between improved band connectivity and stability. These comparisons underscore how anion substitution (X = S, Se, and/or halogen) profoundly influences the electronic structure of Ag–Bi PIMs, providing handles to tailor band dispersion, dimensionality, and defect chemistry for better optoelectronic performance.

In summary, the electronic structure of Ag–Bi PIMs is strongly influenced by factors such as CD, vacancies, halide composition, and ED. Their defect-tolerant nature, combined with the ability to tune electronic properties, makes these materials promising not only for solar cells but also for broader applications in optoelectronics and emerging technologies. Continued computational and experimental efforts will be essential to fully realize their potential.

### 3.2. Electronic dimensionality (ED)

**Fig. 2:**
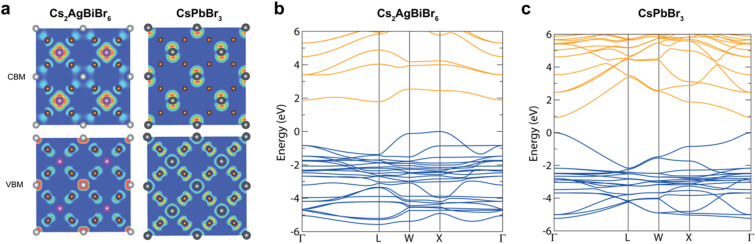
(a) Charge density isosurfaces of the valence band maximum (VBM) and conduction band minimum (CBM), at the Brillouin zone centre. These isosurfaces illustrate the different orbital connectivity in Cs_2_AgBiBr_6_ and CsPbBr_3_. While in the former, the charge density is localised on Bi atoms in the conduction band and on Ag atoms in the valence band, the charge density is more evenly spread in the latter. Ag atoms are shown in light gray, Bi in purple, Pb in dark gray, and Br in brown. Reproduced with permission from ref. 57, licensed under a Creative Commons Attribution (CC-BY) license. (b) and (c). HSE06 + SOC band structures of Cs_2_AgBiBr_6_ and CsPbBr_3_, respectively. Reproduced with permission from ref. 57, licensed under a Creative Commons Attribution (CC-BY) license.

Replicating the superior optoelectronic properties of LHPs—such as narrow bandgaps (<2 eV), absorption coefficients higher than 10^4^ cm^−1^ at the band edge,^48^ high charge-carrier mobilities exceeding 30 cm^2^ V^−1^ s^−1^,^49,50^ and microsecond-long charge-carrier lifetimes^51^—using alternative metal cations remains a formidable challenge. To realize this difficulty, the concept of ED—developed for LHPs and PIMs by Xiao and Yan^52,53^—has gained increasing attention. ED is defined by the degree of orbital connectivity contributing to the valence and conduction bands,^53^ effectively describing how well the orbitals participating in the valence and conduction band edges overlap in all three crystallographic directions. As illustrated in Fig. 2a, high ED originates from strong orbital overlap in 3D networks, while low ED corresponds to limited overlap across 2D layers, one-dimensional (1D) chains, or 0D clusters. While discovering novel PIMs with a high structural dimensionality—*e.g.*, preserving the corner-sharing octahedral network of LHPs—is a core tenet of PIM research, ED has gained increasing attention.^41,54,55^ Importantly, the concept of ED offers a more stringent descriptor than conventional structural dimensionality. Materials can exhibit a 3D structural dimensionality yet possess a low ED due to poor orbital connectivity.^52^

Within this framework, the lower ED of Ag–Bi PIMs is attributed to the combination of two metal cations with distinct electronic configurations: (Bi(iii) with configuration [Xe]4f^14^5d^10^6s^2^ and Ag(i) with configuration [Kr]4d^10^5s^0^).^56^

As described in the previous section, the conduction band edge of Ag–Bi PIMs is generally dominated by Bi 6p orbitals, whereas the valence band edge is mainly composed of halide p orbitals and Ag 4d orbitals (the Bi 6s^2^ lone-pair levels lie deeper in energy and do not set the VBM).^58,59^ This orbital arrangement contributes to a lower ED, which has been thoroughly investigated and demonstrated for Cs_2_AgBiBr_6_. In this material, the weak participation of Ag 5s orbitals results in poor orbital connectivity at the conduction band, leaving the Bi 6p orbital states relatively isolated.^54^ The weak Bi–Ag overlap results in a flat, poorly connected conduction band, contributing to a large electron effective mass and an indirect optical gap. This spatial isolation between conduction and valence band states—localized on [AgX_6_] and [BiX_6_] octahedra, respectively—has been observed across a range of Ag–Bi PIMs (*e.g.*, Cs_2_AgBiBr_6_, AgBiS_2_, and (AgI)_*x*_(BiI_3_)_*y*_).^9,52,54,60,61^ It has been linked to a quasi-0D electronic structure close to the band edges. Consequently, the low ED in these materials has been associated with unfavourable electronic structures (Fig. 2b and c), yielding poor optoelectronic properties such as indirect bandgaps, reduced absorption coefficients (<10^3^ cm^−1^ at the band edge), and low charge-carrier mobilities (<2 cm^2^ V^−1^ s^−1^).^62,63^ Recently, Biega *et al.* further demonstrated that this spatial separation of electron and hole wavefunctions of different cations in Cs_2_AgBiBr_6_ yields localised excited states (*e.g.*, excitons) and has been referred to as a “chemical localisation” effect.^58,64^ Importantly, this localization weakens quantum confinement effects^65,66^—for instance, when transitioning from 3D Cs_2_AgBiBr_6_ to 2D layered PEA_4_AgBiBr_8_—thereby limiting the effectiveness of dimensionality reduction strategies in Ag–Bi PIMs.

To address ED-related limitations, compositional engineering and doping strategies are gaining increasing attention for Ag–Bi PIMs. However, alloying or substituting Ag(i) and Bi(iii) with alternative isoelectronic cations, such as Au(i), Na(i) and Sb(iii), has not yielded significant improvements in the ED,^59,67–69^ reinforcing the idea that the distinct electronic configurations of these ions are a primary cause of low ED in PIMs. In contrast, Hossain *et al.* recently demonstrated that incorporating Ag(i) cations into the 0D Cs_3_Bi_2_I_9_ (A-site compositional mixing) yields a novel 2D semiconductor, Cs_2_AgBi_2_I_9_, with higher ED and improved optoelectronic properties.^8,41^ Similarly, Cu(i) introduction has emerged as a promising route to enhanced ED. While Cu(i) is unsuitable as an A-site cation, several stable Cu-based NaVO_2_-type rudorffites (Cu_4*x*_(AgBi)_1−*x*_I_4_) have been reported by Herz, Snaith and coworkers.^6,55,70,71^ Crucially, the contribution of Cu(i) 3d orbitals to the upper valence band increases the electronic connectivity and reduces the electronic isolation of Ag- and Bi-octahedra.^6,69^ The resulting direct bandgap and low exciton binding energies further underscore the importance of high ED in achieving photovoltaic-relevant properties.^6,71^ Among these, CABI stands out as a particularly promising composition, offering a direct bandgap and a high absorption coefficient of 1.0 × 10^5^ cm^−1^ just above the band edge.^6,72,73^

### 3.3. Charge-carrier transport and optoelectronic properties

The low ED reported for Ag–Bi PIMs impacts not only the quality of these materials as thin-film solar absorbers (*e.g.*, indirect bandgaps, poor absorption coefficients, *etc.*) but also charge-carrier transport.^74,75^ As shown in Table 2, low charge-carrier mobilities (<3 cm^2^ V^−1^ s^−1^) have been consistently reported for Ag–Bi PIMs. Within the Drude model of free charge-carrier conduction, the charge carrier mobility can be defined as *µ* = *eτ*/m*, where *e* is the elementary charge, *τ* is the charge-carrier scattering time and m* is the effective mass of charge carriers.^50^

**Table 2:** Summary of bandgap character, effective masses and electron–hole sum mobilities of prominent Ag–Bi PIMs

Material	Space group	Bandgap character	Effective mass	e–h sum mobility (cm^2^ V^−1^ s^−1^)
CsPbBr_3_	*Fm*3̄*m*	Direct^82^	0.22 (electrons);^83^ 0.24 (holes)^83^	13 (ref. 84)
Cs_2_AgBiBr_6_	*Fm*3̄*m*	Indirect^58,76^	0.33 (electrons);^59^ 0.35 (holes)^59^	3 (delocalized);^85^ 1.3 (localized)^85^
Cu_2_AgBiI_6_ (CABI)	*R*3̄*m*	Direct^6,7^	0.6 (electrons);^6^ 1.0 (holes)^6^	2.1 (delocalized);^71^ 1 (localized)^71^
Cs_2_AgBi_2_I_9_	*P*6_3_/*mmc*	Indirect (quasidirect)^41^	0.6 (electrons);^20^ 2.1 (holes)^20^	36 (ref. 20)
CuAgBiI_5_	*R*3̄*m*	Direct^55^	—	1.7 (ref. 70)
Cu_0.4_AgBiI_4.4_	*R*3̄*m*	Direct^70^	—	0.6 (ref. 70)
Cu_6_AgBiI_10_	*R*3̄*m*	Direct^70^	—	7.3 (ref. 70)
AgBi_2_I_7_		Indirect^86^	—	0.49 (delocalized);^60^ 0.01 (localized)^60^
AgBiI_4_	*R*3̄*m*	Indirect^87^		0.4 (delocalized);^60^ 0.02 (localized)^60^
Ag_2_BiI_5_	*R*3̄*m*	Direct^88^	—	0.49 (delocalized);^60^ 0.03 (localized)^60^
Ag_3_BiI_6_	*R*3̄*m*	Direct^61,89^		0.41 (delocalized);^60^ 0.02 (localized)^60^
AgBiS_2_ (segregated)	*P*3̄*m*1	Indirect^90,91^		0.43 (delocalized);^92^ 0.11 (localized)^92^
AgBiS_2_ (disordered)	*Fm*3̄*m*	Direct^90^	—	2.7 (delocalized);^92^ 2.2 (localized)^92^

**Fig. 3:**
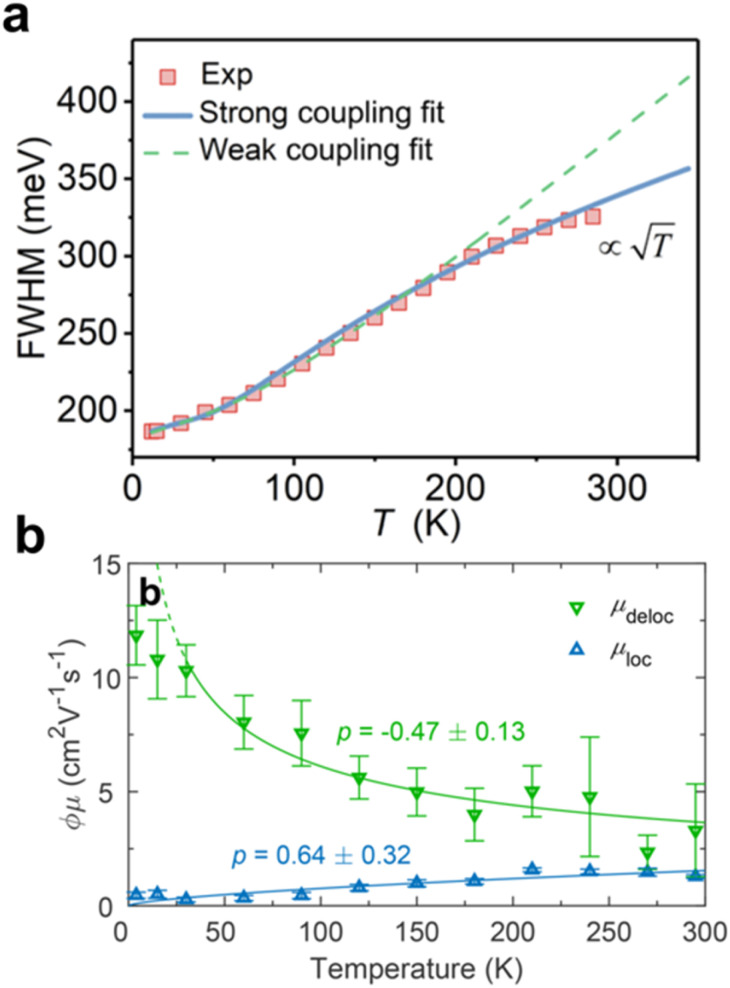
(a) Temperature-dependent photoluminescence (PL) broadening measured for Cs_2_AgBiBr_6_ single crystals. The dashed line indicates the conventional phonon coupling model (weak coupling regime), while the solid line indicates the fit with the Toyozawa strong-coupling model. Reproduced from ref. 80, licensed under a Creative Commons Attribution (CC-BY-NC) license. (b) Temperature-dependent mobility for delocalised (green triangles and line) and localised states (blue triangles and line) in Cs_2_AgBiBr_6_ thin films. Reproduced from ref. 85 under a Creative Commons Attribution (CC-BY 4.0) license.

The low ED of Ag–Bi implies poorer orbital connectivity, which yields less dispersive electronic bands and increased effective masses.^59,76^ However, although higher effective masses were reported for Ag–Bi PIMs with respect to LHPs (see Table 2), these values do not entirely account for the low reported mobilities. Increased charge-carrier scattering rates and a crucial role played by strong electron-phonon scattering have therefore been proposed.^77–79^ In 2021, Wu *et al.* proposed that self-trapping in Cs_2_AgBiBr_6_ is a major intrinsic limitation to the photovoltaic performance of these materials.^80^ Different from early reports indicating predominantly Fröhlich coupling in Ag–Bi halide double perovskites, Wu *et al.* have proposed that a strong coupling with acoustic phonons dominates the electron–phonon interaction *via* the acoustic deformation potential interaction (Fig. 3a).^80,81^ At the same time, Wright *et al.* have reported the presence of an ultrafast (∼ps) decay of photoconductivity in Cs_2_AgBiBr_6_ thin films. As demonstrated by fluence-dependent and temperature-resolved photoconductivity measurements, such decay is also associated with a change in the charge-carrier transport regime from bandlike to hopping transport (Fig. 3b).^85^ Such a swift localization of photogenerated charge-carriers in Cs_2_AgBiBr_6_ not only yields a reduction in charge-carrier mobility—from ∼3 to ∼1 cm^2^ V^−1^ s^−1^—but also imposes a less-efficient hopping transport regime. Crucially, while an energetic barrier to self-trapping is expected for higher-dimensional 3D and 2D semiconductors,^93,94^ the lower dimensionality of the Cs_2_AgBiBr_6_ electronic structure (*i.e.*, its lower ED) has been proposed as a cause of barrierless trapping.^71,85^

Recently, charge-carrier localization processes have been reported for a wide variety of Ag–Bi PIMs, including CABI, (Cu_4*x*_(AgBi)_1−*x*_I_4_), Cs_2_AgBi_2_I_9_, AgBiS_2_, (4FPEA)_4_AgBiI_8_, and (AgI)_*x*_(BiI_3_)_*y*_,^8,60,66,70,71,92,95^ all showing a similar ultrafast loss in charge-carrier mobility. Furthermore, such localization has been reported in analogous systems containing Au(i), Na(i) and Sb(iii)^59,67,68^—showing a similarly low ED—thus further indicating the role played by low ED arising from the combination of d^10^ and s^2^ metal cations.

### 3.4. Cation disorder (CD) and cation vacancies

**Fig. 4:**
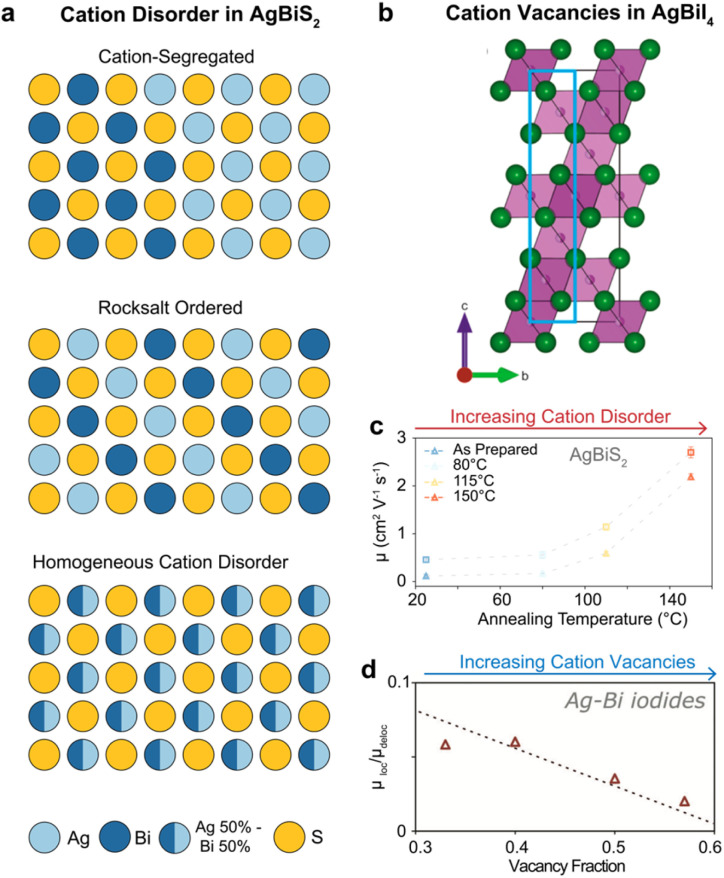
(a) Illustration of different types of cation ordering in AgBiS_2_. The partial occupancy of the Ag(i) and Bi(iii) cation sites is represented as fractional filling of the circles at each site. These schematics of AgBiS_2_ structures depict ideal segregated (top), ordered (middle), and homogeneously disordered cases (bottom). Reproduced from ref. 92, licensed under a Creative Commons Attribution (CC-BY 4.0) license. (b) Illustration of cation vacancies in AgBiI_4_. Adopting the CdCl_2_-type structure, AgBiI_4_ shows a layered structure with alternating fully occupied and entirely vacant layers. Reproduced from ref. 87, licensed under a Creative Commons Attribution (CC-BY) license. (c) Charge-carrier mobility in AgBiS_2_ nanocrystals thin films, as a function of annealing temperature. With increasing temperatures, the increasingly homogeneous CD yields improved charge-carrier mobilities for both delocalised (squares) and localised (triangles) states. Reproduced with permission from ref. 92, licensed under a Creative Commons Attribution (CC-BY 4.0) license. (d) Ratio between the mobility of localised and delocalised charge carriers in (AgI)_*x*_(BiI_3_)_*y*_ thin films as a function of the fractional vacancy concentration in the rudorffite structure. Reproduced from ref. 60 under the terms of Creative Commons Attribution (CC-BY 4.0) license.

Despite their different electronic configurations, octahedrally coordinated Ag(i) and Bi(iii) cations have similar ionic radii, 126 and 119 pm, respectively.^96,97^ This similarity lowers the formation energy of Ag_Bi_ and Bi_Ag_ antisite defects in several Ag–Bi PIMs and promotes a disordered distribution of metal cations in these materials.^9,98^ CD—defined as the occupation of a cation lattice site by a different cation species—has been reported as energetically favourable, and thus likely inevitable, during the synthesis of Cs_2_AgBiBr_6_.^98–100^ Theoretically, CD has been investigated using DFT in various Ag–Bi PIMs (such as Cs_2_AgBiBr_6_ and AgBiS_2_).^90,91,101^ These studies reveal thermodynamic transitions from a fully ordered (rock salt) to a fully disordered cation distribution, with energetic barriers as low as 17 meV.^9^ As illustrated in Fig. 4a, the Ag(i)/Bi(iii) distribution in Ag–Bi PIMs typically comprises a mixture of three configurations: ordered (checkerboard), disordered (equal probabilities of Ag/Bi occupancy of lattice sites), and segregated (Ag- or Bi-rich domains). These different configurations have a profound impact on the optoelectronic properties of Ag–Bi PIMs.^9,54,92,96,98,99^ For instance, significant bandgap narrowing and an indirect-to-direct transition have been reported in Cs_2_AgBiBr_6_,^9,98,99^ attributed to disorder-induced modifications in the band structure. Similarly, Konstantatos and co-workers extensively characterized CD in AgBiS_2_,^9,90,91^ observing both bandgap narrowing and enhanced absorption coefficients. Notably, Wang *et al.* recently demonstrated that a more spatially homogeneous density of states—indicative of a higher ED—in disordered AgBiS_2_ underpins these improved optoelectronic properties.^9^

Beyond the disordered distribution of Ag(i) and Bi(iii) cations, neutral structural vacancies have also been proposed to contribute to the disordered cation distribution in Ag–Bi PIMs.^6,61,70,87,102^ Turkevych *et al.* have described the structure of the Ag–Bi rudorffite series (AgBi_2_I_7_, AgBiI_4_, Ag_2_BiI_5_, and Ag_3_BiI_6_) as an edge-sharing octahedral network, with [MX_6_] octahedra exhibiting fractional occupancy modelled as Ag_*a*_Bi_*b*_Δ_*c*_ (Δ represents a vacancy), where *a*, *b*, and *c* are the fractional occupation ratios.^7^ Similarly, Sansom *et al.* and Buizza *et al.* reported structural vacancies for AgBiI_4_,^87^ CABI,^6^ and within the Cu_4*x*_(AgBi)_1−*x*_I_4_ series (see Fig. 4b).^70^ Importantly, Ag and Bi vacancies can break local symmetries, create local strains, and modulate lattice softness in Ag–Bi PIMs.^12,60^

For instance, Annurakshita *et al.* recently demonstrated that vacancy-induced lattice symmetry breaking can be engineered to tune the NLO properties of Ag–Bi PIMs.^12^

CD and cation vacancies also significantly influence charge-carrier transport in Ag–Bi PIMs. Antisite defects (Ag_Bi_) have been shown to introduce deep trap states that act as non-radiative charge-carrier recombination centres.^101^ Maiti *et al.* further suggested that increased electrostatic repulsion between neighbouring octahedra and lattice distortion occur in disordered Cs_2_AgBiBr_6_.^100^ Cation-segregated configurations (see Fig. 4a) have been linked to the formation of localized states in both AgBiS_2_ and NaBiS_2_.^9,68^ Importantly, Righetto *et al.* demonstrated that charge-carrier localization is mitigated in more homogeneously disordered AgBiS_2_ (Fig. 4c),^92^ highlighting how disorder-induced ED-tuning can help overcome localization effects in Ag–Bi PIMs. Furthermore, Lal *et al.* recently showed a strong correlation between the extent of charge-carrier localization and the abundance of cation vacancies in the lattice (Fig. 4d),^60^ suggesting that vacancy-induced lattice softness may hinder charge-carrier transport. While the study of CD and cation vacancies in Ag–Bi PIMs is still emerging, engineering cation distribution—*e.g.*, through compositional fine-tuning,^12^ thermal annealing,^9,98^ and coordination chemistry^100^—has become a highly promising strategy for optimizing performance in these materials.

### 3.5. Defect chemistry

As detailed in the preceding sections, the defect physics of Ag–Bi PIMs differ substantially from that of conventional LHPs. DFT calculations reveal that most cation-related point defects—such as Ag or Bi vacancies and interstitials—have higher formation energies than their LHP counterparts, indicating intrinsically lower equilibrium defect densities. In contrast, anion vacancies (Br^−^, I^−^, and S^2−^) and the Bi_Ag_ antisite exhibit comparatively low formation energies. The electronic nature of these defects is highly composition-dependent: they tend to be shallow in sulfide and iodide phases but can act as deep recombination centers in Cs_2_AgBiBr_6_ and related bromides. Despite theoretical predictions of limited deep traps from isolated cation defects, Ag–Bi PIMs frequently show weak or broadened PL signals and large *V*_OC_ deficits—clear signs of strong non-radiative recombination. This discrepancy suggests that extrinsic imperfections—such as surface states, halide or chalcogen vacancies introduced during processing, and complex defect clusters—may dominate the recombination landscape. Importantly, the higher oxidation state of Bi(iii), as discussed in the Electronic band structure section, underpins this distinct defect chemistry, providing a critical basis for understanding defect formation and behaviour in these materials. The remainder of this section analyses both experimentally observed and theoretically predicted defect chemistries across representative Ag–Bi compounds, aiming to identify compositions and processing strategies that suppress deep-level defects and enable high-performance, lead-free optoelectronics.

Here, we analyse the defect chemistry of Ag–Bi PIMs and correlate it with device performance metrics where available. We formulate defect-formation reactions using the Kröger–Vink notation for representative Ag–Bi materials, including Cs_2_AgBiBr_6_, Cs_2_AgBi_2_Br_9_, CABI, AgBiI_4_, Ag_2_BiI_5_, AgBiS_2_, and AgBiSCl_2_. This approach helps elucidate the impact of native point defects on carrier dynamics, recombination losses, and optoelectronic performance, ultimately guiding material optimization strategies.

**Fig. 5:**
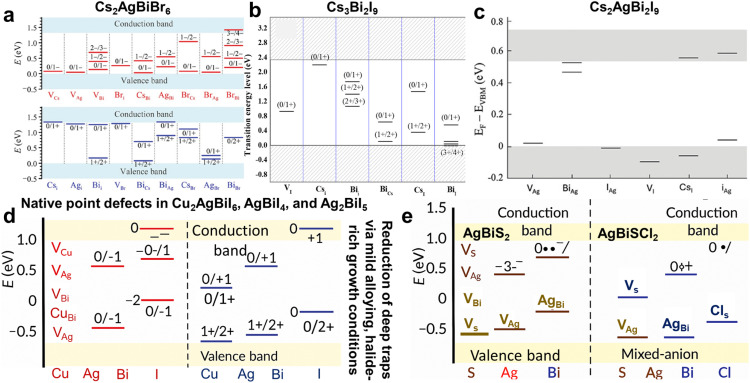
Energy level diagrams and defect energetics in Ag–Bi halides and chalcohalides. (a) Calculated charge transition levels of native point and antisite defects in Cs_2_AgBiBr_6_, including V_Cs_, V_Ag_, V_Bi_, Bi_Cs_, and Ag_Bi_, with their respective charge states across the bandgap. Reproduced with permission from ref. 103. Copyright 2016, WILEY-VCH. (b) Thermodynamic transition levels (*E*_*q*_/*q*′) of intrinsic point defects in Cs_3_Bi_2_I_9_, referenced to the valence band maximum (VBM), showing the depth and recombination potential of each defect. Reproduced with permission from ref. 104. Copyright 2017, American Chemical Society. (c) Distribution of key defect levels in Cs_3_Bi_2_I_9_, with mid-gap states such as V_I_ and Bi_Ag_ expected to act as nonradiative recombination centers. Reproduced with permission from ref. 105 under the terms of the CC-BY license. Copyright 2024. The Author(s), published by Wiley-VCH GmbH. (d) Defect transition levels in Cu_2_AgBiI_6_ (CABI), AgBiI_4_, and Ag_2_BiI_5_, where shallow acceptors like V_Cu_ and V_Ag_ support p-type conductivity, while deeper defects such as V_Bi_, Cu_Bi_, and Ag_Bi_ introduce trap states that degrade carrier lifetimes. (e) Comparative defect chemistry in AgBiS_2_ and AgBiSCl_2_, where V_S_ acts as a dominant deep donor in AgBiS_2_, while V_Ag_, V_Bi_, and Ag_Bi_ serve as acceptor-type defects. In AgBiSCl_2_, halide incorporation introduces additional defects such as V_Cl_ and mixed-anion substitutions (S_Cl_ and Cl_S_), modifying the defect landscape and potentially mitigating deep-level recombination pathways. Panels (d and e) were fabricated primarily based on the reported defect energy level data presented in ref. 70, 106, 107, 92 and 107–109, respectively.

In Cs_2_AgBiBr_6_, bromine vacancies (V_Br_), common due to their low formation energies, act as shallow trap states *via*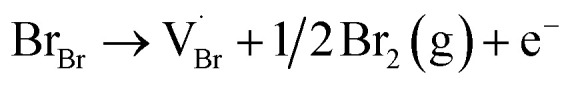 and contribute to radiative recombination. Cesium vacancies (V_Cs_) act as acceptors and induce slight p-type behaviour through Cs_Cs_ → V_Cs_^−^ + Cs (g or sol). Silver vacancies, V_Ag_, similarly generate holes: → Ag_Ag_ → V_Ag_ + Ag (g or sol). Antisite defects like Ag_Bi_ (silver on a bismuth site) and Bi_Ag_ (bismuth on a silver site) introduce deep trap states that enhance nonradiative recombination and lattice distortion formed by Ag_Ag_ + Bi_Bi_ → Ag_Bi_ + Bi_Ag_. Bromine interstitials (Br_i_), though less frequent due to high formation energy, form under Br-rich conditions and follow 
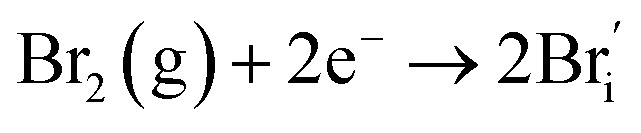
, increasing the ionic conductivity. Device performance is primarily limited by deep traps from Ag_Bi_ and Bi_Ag_, as shown by DFT calculations (Fig. 5a).^110^ Controlling growth conditions is key; Xiu *et al.* found that Ag-rich and Br-poor conditions optimize n-type conductivity and suppress secondary phases.^111^

In Cs_3_Bi_2_I_9_, iodine vacancies—one of the most common and energetically favourable defects—form *via* the reaction 
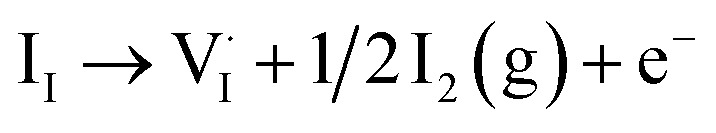
, creating positively charged traps that promote nonradiative recombination. Cesium vacancies act as acceptors and lead to p-type conductivity, following the reaction Cs_Cs_ → V_Cs_^−^ + Cs(g). Bismuth vacancies, though less favorable energetically, can also act as acceptors *via* Bi_Bi_ → V_Bi_^3−^ + Bi(g). Antisite defects such as Bi_Cs_ and Cs_Bi_ result in local lattice distortions and deep-level trap states, represented by the reaction Bi_Bi_ + Cs_Cs_ → Bi_Cs_^2+^ + Cs_Bi_^2−^. Alloying Ag^+^ at the Cs^+^ site offers a promising defect mitigation strategy (Fig. 5b).^112^ First, Ag^+^ forms stronger Ag–I bonds than Cs–I, thereby stabilizing iodide ions and suppressing iodine vacancy formation; the reaction 
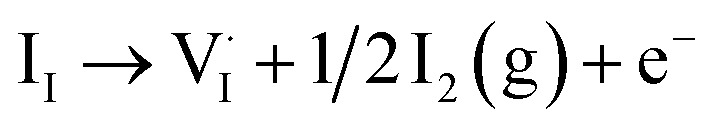
 becomes less favorable in the presence of Ag. Second, Ag^+^ substitution reduces Cs vacancy formation due to site occupancy, as described by Ag_Cs_ + Cs_Cs_ → Ag_Cs_^0^ + V_Cs_^−^ + Cs(g), making V_Cs_ generation energetically less favourable (Fig. 5c).^105^ Lastly, Ag^+^ ions tend to avoid substituting for Bi^3+^ sites, which limits the formation of antisite defects. This selective site preference helps preserve the crystal structure and minimizes the creation of mid-gap electronic states, thereby enhancing the optoelectronic performance of the material.

The defect chemistry of CABI, AgBiI_4_, and Ag_2_BiI_5_ is governed by native point defects, with cationic vacancies at the Cu^+^ and Ag^+^ sites playing a dominant role in determining their optoelectronic properties. In CABI, copper vacancies (V_Cu_) are the most energetically favourable acceptor-type defects, contributing to p-type conductivity *via* the reaction Cu_Cu_ → V_Cu_^−^ + Cu(g), and these shallow acceptors aid hole transport with minimal nonradiative recombination. Likewise, silver vacancies (V_Ag_), which are common across CABI, AgBiI_4_, and Ag_2_BiI_5_, form through the reaction Ag_Ag_ → V_Ag_^−^ + Ag(g) and also contribute to p-type conductivity, though at high concentrations they may introduce instability or recombination losses (Fig. 5d).^16,70,113^ Iodine vacancies (V_I_), which act as deep-level donor defects, are formed *via*
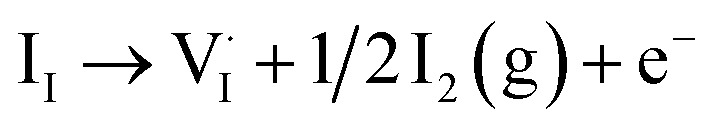
, and are detrimental as they promote nonradiative recombination and degrade photovoltaic performance. In Ag-rich phases such as AgBiI_4_ and Ag_2_BiI_5_, antisite defects (*e.g.*, Bi_Ag_ and Ag_Bi_) can arise due to charge and size mismatches, creating deep traps and inducing local lattice distortions. While CABI largely tolerates CD, precise stoichiometry and defect passivation—*via* mild alloying or halide-rich growth—are key to reducing deep defects and improving carrier lifetimes, stability, and performance.

The defect chemistry of AgBiS_2_ and AgBiSCl_2_ is primarily governed by intrinsic point defects, with sulfur vacancies (V_S_) being the most prevalent due to the relatively weak Ag–S and Bi–S bonds, particularly under sulfur-poor or high-temperature conditions. These sulfur vacancies act as donor-type defects, forming *via* the reaction 
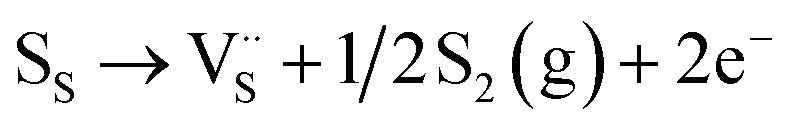
, where 
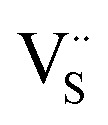
 denotes a doubly positively charged vacancy and the released electrons contribute to n-type conductivity. However, these defects introduce deep-level trap states that enhance nonradiative recombination and degrade carrier transport. In AgBiS_2_, silver vacancies (V_Ag_) commonly form under Ag-deficient conditions through the reaction Ag_Ag_ → V_Ag_ + Ag(g), contributing to p-type behaviour, while bismuth vacancies (V_Bi_^3−^) may form *via* the reaction Bi_Bi_ → V_Bi_^3−^ + Bi(g), though with higher formation energy due to Bi's stronger bonding. Additionally, antisite defects such as Ag_Bi_^2+^ and Bi_Ag_^2−^ can emerge through Ag_Ag_ + Bi_Bi_ → Ag_Bi_^2+^ + Bi_Ag_^2−^, inducing local distortions and deep traps (Fig. 5e).^92,114–116^ In AgBiSCl_2_, the presence of highly electronegative Cl^−^ modifies the defect landscape by partially stabilizing the lattice and suppressing V_S_ formation, although sulfur vacancies can still form under S-deficient conditions by the same reaction. Cl vacancies (V_Cl_) may also appear *via* the reaction 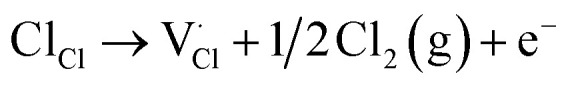, acting as shallow donors. Furthermore, mixed-anion defects such as S_Cl_^−^ and Cl_S_^+^, arising from site exchange reactions (S_S_ + Cl_Cl_ → S_Cl_^−^ + Cl_S_^+^), can significantly perturb the electronic structure and affect charge transport. AgBiS_2_ displays strong n-type behaviour with sulfur vacancies, while AgBiSCl_2_ has more complex defect chemistry with V_S_, V_Cl_, and anion substitutions. Optimizing the growth conditions (sulfur-rich for AgBiS_2_ and Cl-rich for AgBiSCl_2_) and controlling the Ag : Bi ratio are crucial for reducing recombination losses and improving performance.

In summary, AgBiS_2_, AgBiSCl_2_, and CABI possess lattices with greater defect tolerance than many other Ag–Bi PIMs, resulting in fewer intrinsic deep traps and, consequently, enhanced optoelectronic device performance. In contrast, AgBiI_4_ and Cs_2_AgBiBr_6_ suffer from abundant deep trap states and Ag/Bi antisite defects that significantly impair the device efficiency. Whether these intrinsic differences ultimately limit—or can be engineered to enhance—optoelectronic performance depends on how charge carriers interact with both the defect landscape and the soft, highly polarizable lattice. The following section moves from static defect energetics to their dynamic consequences, briefly examining how carrier trapping, small-polaron formation and exciton binding energies shape the observable photophysics of the Ag–Bi family.

### 3.6. Defect mitigation in Ag–Bi PIMs

Defect passivation in Ag–Bi PIMs has become one of the most critical areas to facilitate its application in efficient optoelectronics, as the intrinsic defects strongly affect carrier recombination, stability, and material efficiency in general. A recurring motif in the literature is that the growth conditions can be tuned to reduce the number of harmful point defects, such as halogen vacancies and cation antisites, that are known to be the primary non-radiative recombination centers. For instance, halide stoichiometry adjustment in the Cs_2_AgBiBr_6_ synthesis process significantly minimizes bromine vacancies. At the same time, the Ag : Bi ratio control suppresses antisite defects like Ag_Bi_ and Bi_Ag_, leading to enhancement in both stability and optoelectronic quality.^117,118^ Alloying approaches also offer a promising means to neutralize deep traps and stabilize lattice environments; incorporation of Ag^+^ into Cs_3_Bi_2_I_9_ has been demonstrated to reinforce Ag–I interactions and inhibit iodine vacancy generation, while mild alloying with trace amounts of heterovalent or isovalent cations induces local strain fields that passivate shallow states and prolong carrier lifetimes.^8,20^ In addition to intrinsic defect control, extrinsic defect passivation strategies, including surface functionalization or post-synthesis annealing, provide further means of control, with sulfur-rich annealing of AgBiS_2_ as a prototypical system where defect healing directly enhances carrier transport.^119,120^ At the processing stage, sophisticated synthesis techniques—like growth under controlled inert atmospheres—can mitigate oxidation-induced defects in materials such as AgBiSCl_2_, and thorough growth temperature optimization can reduce deep-level state formation in sulfide materials. Real-time feedback is increasingly being integrated into synthesis through advanced characterization techniques, as *in situ* monitoring allows for dynamic modification of growth parameters, and defect mapping through PL imaging reveals local defect concentrations to inform subsequent optimization pathways. Concurrent work in compositional engineering reveals that the addition of stabilizing anions, such as chloride in AgBiSCl_2_, modifies the defect landscape to favour higher electronic conductivity, and mixed-anion approaches (*e.g.*, S^2−^/Cl^−^ co-substitution) lead to beneficial changes in the electronic structure that may suppress non-radiative processes. Significantly, the defect chemistry engineering towards shallow traps is an effective approach to enhance radiative recombination, and the higher halide content in Cs_2_AgBiBr_6_ leads to shallow trap states, which significantly improve photoluminescence and charge transport. Collectively, these advances provide an integrated toolbox for defect control in Ag–Bi PIMs, spanning growth optimization, alloying, passivation, processing control, and compositional engineering that can be viewed as a unified approach to performance enhancement. However, although progress has been made, challenges remain in relating defect formation energetics to full-device stability under operational stress, underscoring the need for systematic, long-term investigations that integrate computational defect modelling with *in situ* experimental verification. With this knowledge, the community can progress towards design rules for the rational design of defect-tolerant Ag–Bi PIMs, which will enable their use as a robust, lead-free option for application in next-generation solar and optoelectronic technologies.

### 3.7. Photophysical properties

The photophysical performance of Ag–Bi PIMs, such as Cs_2_AgBiBr_6_, Cs_2_AgBi_2_I_9_, and AgBiS_2_, is often hindered by the above-discussed intrinsic limitations—defect-related losses, carrier trapping, small polaron formation, and high exciton binding energies, primarily governed by their intrinsic defect chemistry and low ED.

The low-dimensional crystal or electronic structure yield pronounced excitonic effects, evident as distinct exciton resonances in absorption spectra. As a result, very high exciton binding energies have been consistently reported for Cs_2_AgBiBr_6_ (>200 meV) as well as AgBiI_4_ and Ag_2_BiI_5_ (>150 meV)—significantly exceeding those of LHPs (∼25 meV for MAPbI_3_)—severely limiting efficient charge separation at room temperature.^121,122^ However, for CABI, a remarkably low exciton binding energy of approximately 25 meV was initially reported (estimated by the Elliott fitting of absorption spectra),^6^ suggesting efficient free-carrier generation. However, a more recent study reported a considerably higher exciton binding energy (>100 meV), which was reduced to ∼100 meV through partial Sb^3+^ incorporation.^45^ Another recently discovered material, Cs_2_AgBi_2_I_9_, has been found to exhibit a relatively low exciton binding energy (∼40 meV),^8^ consistent with the enhanced ED discussed previously. These findings highlight how targeted compositional tuning is a crucial strategy to mitigate exciton-related limitations in Ag–Bi halide PIMs and thereby enhance their optoelectronic performance. AgBiS_2_ is expected to exhibit a high exciton binding energy, especially in its quantum dot form, due to enhanced quantum confinement effects.

In fully 3D-ordered double perovskites like Cs_2_AgBiBr_6_, the fundamental bandgap is indirect (∼2.2 eV), with the first allowed optical transition being at higher energy (∼3.0 eV). Consequently, this shows a weak absorption onset, with strong absorption emerging only at higher-energy direct transitions.^123^ In contrast, solution-processed direct bandgap CABI films achieve ∼1 × 10^5^ cm^−1^ near the band edge, due to allowed direct transitions.^6^

**Fig. 6:**
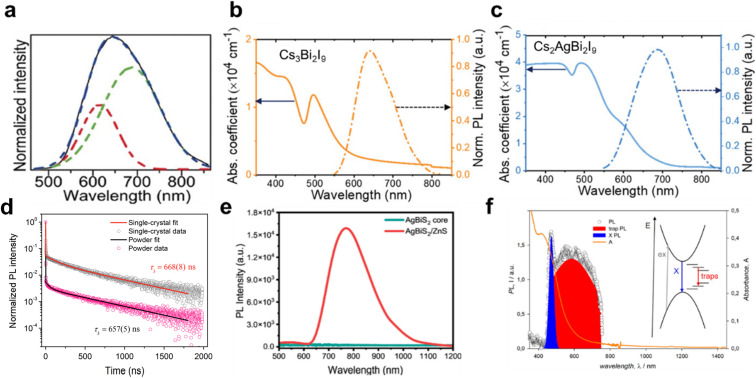
Representative absorption and photoluminescence (PL) characteristics of Ag–Bi PIMs. (a) Deconvoluted PL spectrum showing multiple recombination channels in a representative Ag–Bi PIM, Cs_2_AgBiBr_6_. Reproduced with permission from ref. 127. Copyright 2016, WILEY-VCH. (b) Absorption coefficient and normalized PL intensity of Cs_3_Bi_2_I_9_, highlighting low absorption near the band edge and broad PL emission. (c) Optical properties of Cs_2_AgBi_2_I_9_, including absorption onset and broad PL emission, indicating sub-bandgap recombination. Panels (b and c) are reproduced with permission from ref. 105 under the terms of the CC-BY license. Copyright 2024. The Author(s), published by Wiley-VCH GmbH. (d) Time-resolved PL decay curves of single crystal and powder samples of Cs_2_AgBiBr_6_. Reprinted (adapted) with permission from ref. 125. Copyright 2016, American Chemical Society. (e) PL comparison between the AgBiS_2_ core and AgBiS_2_/ZnS core–shell nanocrystals, revealing strong PL enhancement upon surface passivation with ZnS, indicating the suppression of surface trap states. Reproduced from ref. 47 under the terms of CC-BY 4.0. Copyright 2024, American Chemical Society. (f) Steady-state PL spectrum of AgBiSCl_2_ NCs (inset: schematic representing the origin of the trap-assisted PL component of the PL spectrum of the NCs). Reproduced from ref. 10 under the terms of CC-BY 4.0. Copyright 2023. The Author(s), published by American Chemical Society.

Nonetheless, low ED usually broadens PL spectra and suppresses PL quantum yields, since photo-excitations tend to localize. In Cs_2_AgBiBr_6_, a broad red PL band peaking around 600–700 nm (1.9–2.0 eV) is observed at room temperature (Fig. 6a). This emission is strongly Stokes-shifted from the absorption edge and was initially attributed to an indirect exciton transition or sub-gap defect emission, but now known to originate from self-trapped excitons (STEs) and small polarons *via* advanced calculations and spectroscopic studies.^85,123^

Cs_3_Bi_2_I_9_ exhibits strong carrier localization and weak PL (Fig. 6b) due to deep defects and an indirect gap (∼2.0–2.2 eV); incorporating Ag^+^ to form Cs_2_AgBi_2_I_9_ slightly improves the dimensionality and reduces the bandgap (∼1.8 eV) but still displays emission characteristics due to polaron-induced effects (Fig. 6c).^8^ The appearance of a shoulder peak in the absorption spectrum after Ag incorporation causes the reduced bandgap in Cs_2_AgBi_2_I_9_ compared to Cs_3_Bi_2_I_9_.

(Cu)–Ag–Bi–I systems, such as CABI and (AgI)_*x*_(BiI_3_)_*y*_, exhibit ultrafast self-localization of charge carriers, characterized by picosecond-scale conductivity losses and broad, weak, redshifted PL,^60,71,95^ alongside additional non-radiative recombination pathways linked to Cu/Ag vacancies and antisite defects. Such self-trapping arises from strong coupling between charge carriers and lattice distortions (high deformation potentials) associated with electronically soft, low-dimensional lattices. Photoexcited carriers in many Ag–Bi PIMs typically localize on ultrafast (sub-picosecond) timescales. Time-resolved studies have shown carrier self-localization rates around 1–2 ps, along with similarly barrierless, sub-picosecond trapping in AgBiS_2_ nanocrystals.^60,71,85,124^ However, homogeneous CD in AgBiS_2_ notably reduces the extent of charge-carrier localization.^124^ Conversely, Sb incorporation into CABI leads to broader STE emission, indicative of enhanced carrier self-trapping due to structural modifications. This reduces trap-mediated recombination, as evidenced by transient absorption studies, ultimately improving the photovoltaic performance.^45^

Since the ultrafast self-trapping driven PL tends to be broad and of low intensity in which case radiative recombination occurs from a distorted, lower-energy state, the STE PL in Ag–Bi PIM lifetimes often becomes multi-exponential, with a dominant short component from non-radiative traps and a long-lived tail (up to hundreds of nanoseconds or even microseconds) from radiative STE recombination. Single crystals of Cs_2_AgBiBr_6_, for instance, show a long-lived PL component of approximately 690 ns (Fig. 6d), explained by slow radiative decay of STEs despite a high defect density.^125^ However, a combination of ultrafast self-trapping and trapping at the surface and bulk of the carriers in polycrystalline limited the diffusion lengths to just 30 nm and mobilities to <1 cm^2^ V^−1^ s^−1^.^126^

High defect concentrations and deep trap states (common in these materials) contribute to the lack of band-edge PL and weak STE PL intensities, funnelling carriers into non-radiative paths. Intrinsic defect chemistry in Ag–Bi PIMs is complex, as these compounds readily form vacancies and antisite defects that introduce deep recombination centres. Without deliberate passivation, non-radiative recombination *via* such defects is a major loss pathway, severely reducing PL quantum yields and carrier transport properties. For AgBiS_2_ nanocrystals, coating with a ZnS shell induces near-infrared PL emission at 764 nm (Fig. 6e) by passivating surface defects (Ag/S vacancies and antisites), enhancing carrier confinement through a type-I band alignment, and suppressing small-polaron forssmation and ultrafast trapping.

Interestingly, AgBiSCl_2_ NCs exhibit PL originating from both band edge excitons and midgap states—marking the first report of band edge emission in chalcohalide nanomaterials—where the partial suppression of self-trapping and a more favourable electronic structure enable radiative recombination, although non-radiative decay *via* midgap states introduced by Cl vacancies, disorder, and antisites still dominates (Fig. 6f).

In summary, the photophysical features of Ag–Bi PIMs are strongly influenced by their low ED, intrinsic defects, and pronounced electron–phonon coupling. These characteristics result in substantial exciton binding energies, rapid carrier self-localization into small polarons, limited mobility, and weak, broad PL. Strategies such as compositional tuning (*e.g.*, Sb incorporation into CABI), CD engineering (as in the case of AgBiS_2_), and defect passivation (*e.g.*, ZnS coating on AgBiS_2_ nanocrystals) effectively mitigate these limitations, highlighting pathways toward improved optoelectronic performance in this intriguing family of materials.

### 3.8. Thin-film deposition: recent progress and persistent challenges

A major challenge for Ag–Bi PIMs is producing uniform, compact, impurity-lean films under scalable conditions. Unlike defect-tolerant Pb perovskites, several Ag–Bi phases show performance tightly coupled to the microstructure and deep-trap/impurity chemistry, so deposition windows (temperature, halogen fugacity, and nucleation kinetics) must be controlled more narrowly to suppress CuI/AgI domains or vacancy complexes. Recent studies across the families clarify where these windows lie and which process controls matter most. Broad surveys emphasize that, while the materials base has diversified, advances in annealing/antisolvent control and precursor/additive chemistry now govern the film quality as much as the choice of toolset (solution *vs.* vacuum).^106,128^

For Cs_2_AgBiBr_6_,^129–134^ solution films benefit from antisolvent-assisted nucleation (*e.g.*, IPA and methyl acetate) and hot-casting/gas-quenching, which promote dense, ultra-smooth coverage; the first inverted devices (2.23%, *V*_OC_ ≈ 1.01 V) were realized using IPA drip + high-T anneal, and subsequent work shows that post-annealing ≥250–285 °C is often required for phase-pure films from solution. Optimizing preheat and anneal setpoints (and the timing of the antisolvent drop) consistently improves the grain size and crystallinity. Dry/semidry methods (pulsed layer deposition and chemical vapor deposition) add scalability by tuning the deposition pressure/temperature for highly crystalline layers. Even so, the current record 6.37% stems from hydrogenation of spin-cast films, which narrows *E*_g_ from ≈2.18 eV to ≈1.64 eV and passivates halide-vacancy traps—evidence that defect energetics, not morphology alone, set the efficiency ceiling.

For Cu–Ag–Bi–I—especially CABI—the thin-film picture diverges by route. Co-evaporation is highly sensitive to the anneal temperature (≈110–150 °C) and iodine fugacity: modest increases drive CuI-rich impurity domains, visible as a sharpened ≈415 nm absorption shoulder and a strong ≈730 nm PL band.^33^ These domains shorten electron-transport lengths and can produce deceptively bright PL while degrading the device current (front/back EQE measurements reveal electron-extraction bottlenecks in planar cells prepared under such conditions).^33^ In contrast, solution processing has yielded repeatable improvements *via* additive and solvent engineering: hydroiodic acid (HI) additions increase the surface coverage and reduce interfacial recombination (early devices ≈1.3% AM 1.5G),^135^ while hypophosphorous acid (H_3_PO_2_) generates *in situ* Ag nanoparticles and strengthens Bi–(H_3_PO_2_/H_3_PO_3_) coordination, giving smooth films (RMS ∼24 → 14 nm), lowered ideality factor (1.64 → 1.15) and improved V_bi_ (0.81 → 0.86 V). These CABI devices delivered improved photovoltaic performance.^136^ Cation engineering (partial Sb^3+^ alloying on the Bi site) also reduced trap densities and delivered a 1-sun PCE of 1.82%,^45^ while halide engineering tuned lattice/defect chemistry in mixed-iodide/bromide analogues.^43^ In short, co-evaporation demands a narrow thermal window, whereas solution routes profit from controlled chemistry for uniformity and reduced non-radiative recombination loss.

For AgBiS_2_ (chalcogenide),^9,11,137^ the decisive control is densification. Vapor-assisted solution and chemical vapor deposition/co-evaporation now produce sub-micron-grain, pinhole-free films with 10.20% (0.06 cm^2^) and 9.53% (1.00 cm^2^) PCE and strong durability (*e.g.*, ≥94% retained after ∼3000 h ambient, ≈87% after 1000 h at 85 °C). Parallel progress in nanocrystal inks shows that post-deposition *in situ* passivation (*e.g.*, ligand exchange/halogenated agents) can make ultrathin, trap-lean films with FF ≈ 72% and >10% PCE. Solution-crystallized “thick” films deliver *J*_SC_ > 31 mA cm^−2^ under AM 1.5G. Thermal co-evaporation has also yielded phase-pure AgBiS_2_, underscoring that both vacuum and solution tracks can meet the film-quality bar when voiding/cracking are suppressed by proper rheology and drying-kinetics control.

While AgBiS_2_ has been synthesized in nanocrystal form (limited synthesis efforts so far),^10^ other Ag–Bi–I materials (*e.g.*, AgBiI_4_, Ag_2_BiI_5_, and AgSb_2_I_7_) are gaining process-specific insights. Rapid thermal/microwave iodization of stacked metal-halide precursors produces AgSb_2_I_7_ thin films,^138^ while data-driven optimization (self-driving labs) has screened >1700 synthesis conditions for AgBiI_4_ in minutes,^139^ yielding pin-hole-free films with larger grains than historical baselines. These show that precursor sequence, iodization kinetics, and anneal ramps are equally central to reproducible morphology in emerging Ag–Bi–I systems.

## 4. Applications

Having established the fundamental photophysical behaviours of Ag–Bi PIMs—characterized by low ED and high CD, strong electron–phonon coupling, and complex defect chemistry—this section now explores how these intrinsic properties influence the behaviour of these semiconductors in practical device applications. We systematically and briefly review recent progress in photovoltaics, photocatalysis, sensing, energy storage, radiation detection, and NLO, highlighting specific strategies employed to overcome inherent limitations and capitalize on the unique multifunctional potential of Ag–Bi-based materials.

### 4.1. Photovoltaics

Ag–Bi PIMs encompass halides, mixed-anion chalcohalides, and fully chalcogenide compounds—a chemical progression that progressively narrows the optical bandgap and raises the absorption coefficient (*α* ≥ 10^5^ cm^−1^), thereby boosting photocurrent in devices under sunlight. Photovoltaic devices based on Ag–Bi PIMs, in general, exhibit significant losses in *V*_OC_ and short-circuit current density (*J*_SC_) that stem from limited defect tolerance, polaron self-trapping, and high exciton binding energies, as explained in the previous sections.

**Fig. 7:**
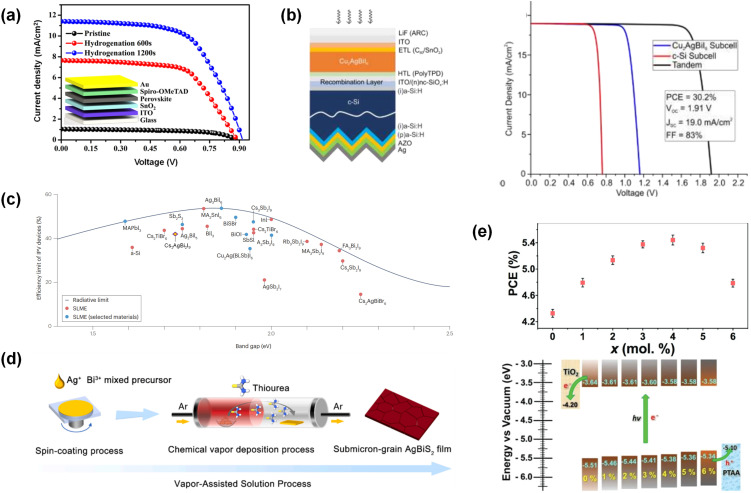
(a) Current density–voltage (*J*–*V*) curves of Cs_2_AgBiBr_6_ photovoltaic cells under 1-sun illumination, comparing samples with varying hydrogenation times (0, 600, and 1200 s). Reproduced from ref. 134 under the terms of the CC-BY license. Copyright 2022. The Author(s), published by Springer Nature. (b) Simulated *J*–*V* curves of a Cu_2_AgBiI_6_/*c*-Si tandem solar cell, calculated using a transfer matrix optical model coupled with detailed balance. The schematic structure of the modelled solar cell is shown alongside. Reproduced from ref. 6 under the terms of the CC-BY 4.0 license. Copyright 2021. The Author(s), published by American Chemical Society. (c) Calculated radiative-limited indoor maximum efficiencies (i-SLME) for selected PIMs, alongside MAPbI_3_ and a-Si, under standard white LED illumination, demonstrating their potential for indoor photovoltaic applications. Data for Cs_2_AgBi_2_I_9_ were estimated under similar assumptions and included as an additional reference point. Reproduced from ref. 140, Copyright 2024, with permission from Elsevier. (d) Schematic illustration of the vapor-assisted solution deposition process used to form AgBiS_2_ absorber layers, enabling solar cells with efficiencies exceeding 10%. Reproduced from ref. 11 with permission from Springer Nature, 2025. (e) Top panel: Power conversion efficiency (PCE) of Au|PTAA|Ag_3_BiI_6−2*x*_S_*x*_|*m*-TiO_2_|*c*-TiO_2_|FTO solar cells as a function of sulfide modification level (*x*). Bottom panel: illustrates the evolution of electronic energy levels as a function of composition (*x*). For reference, the conduction band edge energy of TiO_2_ and the highest occupied molecular orbital (HOMO) level of PTAA are also indicated. Valence band edge positions were determined experimentally using photoelectron spectroscopy in air (PESA). Reproduced from ref. 141. Copyright 2021, published by Wiley-VCH.

The most extensively studied Ag–Bi halide PIM for solar cells is Cs_2_AgBiBr_6_ double perovskite. Its wide bandgap (∼2.2 eV) is sub-optimal for single-junction photovoltaics, initially limiting PCEs to ≈1–2%. Optimised antisolvent casting, ammonium-halide surface passivation and molecular-dye sensitisation have pushed outdoor efficiencies to ≈4.5%.^5^ Under 1000 lux white-LED illumination, the same architecture delivers ≈7.2% indoor PCE.^142^ Deep bromine-vacancy traps drive non-radiative recombination. Low-temperature hydrogen plasma treatment mitigates these traps, narrows the bandgap to 1.64 eV and raises the record outdoor PCE to 6.3% with *V*_OC_ ≈ 0.92 V—currently the highest for any Ag–Bi halide PIM.^134^ With a bandgap of 1.64 eV, the detailed-balance (Shockley–Queisser) limit predicts a *V*_OC_,_SQ_ ≈ 1.35 V at 300 K;^143^ the champion 6.3% Cs_2_AgBiBr_6_ solar cell, which delivers *V*_OC_ ≈ 0.92 V (Fig. 7a); the resulting voltage deficit (Δ*V* ≈ 0.43 V) still signals significant non-radiative loss pathways. Most other Ag–Bi PIM solar cells show even larger Δ*V* values, underlining the need for more aggressive passivation strategies.^11,17^

In Ag–Bi iodide absorbers, solar cell performance correlates strongly with composition. Bismuth-rich phases (with many inherent cation vacancies) show lower PCEs, whereas silver-rich phases (fewer vacancies) generally perform better. The most Ag-rich known compound—Ag_3_BiI_6_—achieved ∼4.3% PCE in 2018.^7^ Nevertheless, its absorption tail (indicating an Urbach energy ≈90 meV) reveals band-edge disorder linked to CD, strong electron–phonon interaction, and high density of traps.^89^

The quaternary iodide CABI (∼2.0 eV bandgap) initially yielded only <1% PCE in thin-film solar cells.^135^ Through improved crystallization, compositional tuning, and interface engineering, recent devices have reached ∼2–3% efficiency.^136,144,145^ Transient absorption and photothermal deflection spectroscopy reveal broad sub-gap absorption to 1.25 eV, confirming deep traps.^6^ These defects severely limit the *V*_OC_ and carrier diffusion lengths. While theory suggests a CABI top cell could reach *V*_OC_ ≈ 1.92 V and ∼30.2% efficiency (Fig. 7b) in tandem with silicon^6^ and >30% PCE under indoor illumination (1000 lux),^140^ realizing those values will require suppressing cation-site disorder, as indicated by the high Urbach energy (≈50 meV).^145^

Cation-alloying effects are composition-dependent. In Cs_2_AgBiBr_6_, substituting Sb^3+^ for Bi^3+^ narrows the bandgap slightly (to ∼2.08 eV at 90% Sb) but induces substantial disorder and deep defect states. The substitution caused quenching PL and added a 0.7 eV sub-gap feature, contributing to 0.7 V *V*_OC_ loss and 120 meV Urbach energy, indicating severe band-tail disorder.^146^ By contrast, partially alloying Sb into CABI slightly reduces its Urbach energy (from 49.6 to 47.6 meV) and boosts the champion PCE from 1.31% to 1.82% (∼40% relative improvement),^145^ showing that judicious alloying can improve defect tolerance. Bulk KSCN passivation has likewise lowered Urbach energy from 120 to 100 meV in AgBiI_4_, signalling improved lattice order.

An intriguing Ag–Bi–I compound, Cs_2_AgBi_2_I_9_, formed by partially substituting Ag into Cs_3_Bi_2_I_9_, displays extended Ag–I–Bi networks that enhance ED. State-of-the-art Cs_2_AgBi_2_I_9_ devices deliver ≈3% outdoor PCE and ≈8% indoor PCE at 1000 lux—record value for Ag–Bi iodides.^8,17^ Although its wide bandgap of ≈1.8 eV would allow >40% indoor PCE, the present low *V*_OC_ values of the devices (∼0.7 V) attest to the residual CD and defect-assisted recombination.^8^ Similar to Cs_2_AgBi_2_I_9_, many wide-bandgap Ag–Bi halide PIMs possess potential indoor PCEs in the 30–50% range (Fig. 7c). Following the emergence of Cs_2_AgBi_2_I_9_, computational studies suggest partial A-site substitution as a potential strategy to address CD in Ag–Bi iodides.^147^ For example, incorporating Cs^+^ into the CABI lattice or partially replacing Ag with Cu in the Cs_3_Bi_2_I_9_ structure could help stabilize the lattice structure and further narrow the bandgap. Such targeted alloying approaches may provide avenues for optimizing the Cu–Ag–Bi–I family's future optoelectronic performance.

When moving to chalcogenides, absorption is pushed towards the near-infrared. Owing to cation-disorder engineering, AgBiS_2_ exhibits an extraordinarily high absorption coefficient of *α* > 2 × 10^5^ cm^−1^ at 500 nm.^9^ Colloidal quantum dot devices of only 30 nm thickness collect ≈27 mA cm^−2^ and reach 9.2% PCE (8.85% certified).^9^ Vapour-assisted crystallisation (Fig. 7d) recently produced ≥10% PCE from polycrystalline films—the highest Ag–Bi PIM efficiency to date.^11^ The Se analogue AgBiSe_2_ has achieved ≈2.6% PCE in quantum dot form,^46^ and continuous-alloy AgBiS_*x*_Se_1−*x*_ enables bandgap tuning across 1.0–1.3 eV.^46^ Collectively, these emerging chalcogenides broaden the horizons for lead-free photovoltaics, pairing high optical performance with robust stability inherent to Ag–Bi materials.

Introducing chalcogenides (S^2−^ and Se^2−^) into Ag–Bi halides further tunes the band structure, enhancing absorption spectra and narrowing the bandgap. A clear example is the sulfur-alloyed double perovskite Cs_2_AgBiBr_6−2*x*_S_*x*_, where adding just ∼3% sulfur significantly improved visible-light harvesting, yielding a PCE around 2% (1.3% for the pristine halide PIM).^148^ Computational and experimental work indicates that heavy chalcogen substitution can induce local lattice distortions and disorder, increasing Urbach tails.^149^ Thus, precise synthetic control is essential to obtain uniform chalcohalide phases. Even partial incorporation of sulfur can greatly enhance the performance: a recent mixed Ag–Bi–I–S thin film (*E*_g_ ∼1.8 eV) achieved 5.6% PCE (Fig. 7e)—the highest among Ag–Bi chalcohalides.^141^ Furthermore, recent computational studies predict that AgBiSCl_2_ (direct *E*_g_ > 2 eV) could surpass 10% PCE under sunlight. In fact, its wide bandgap is more suitable for indoor light harvesting.^10^ Overall, chalcogen-halide alloying emerges as a versatile route to strengthen absorption, suppress deep traps, and bridge the performance gap between pure halide and pure chalcogenide Ag–Bi absorbers.

In summary, Ag–Bi PIMs have progressed from proof-of-concept photovoltaic absorbers to double-digit-efficiency devices within a decade, yet their full potential remains untapped. Continued advances in defect passivation, compositional tuning, and interface engineering^150^ are poised to minimize the remaining voltage and current losses, positioning these lead-free systems as viable contenders for the next-generation of air-stable and environmentally benign photovoltaics.

### 4.2. Photocatalysis and photoelectrocatalysis

Photocatalysis (PC) and photoelectrocatalysis (PEC) are sustainable approaches for driving chemical reactions using light energy, offering potential in applications like water splitting, CO_2_ and N_2_ reduction, and pollutant degradation. Ag–Bi PIMs have emerged as promising candidates due to their unique optoelectronic properties, such as strong light absorption, suitable band structures, and intrinsic stability.^151^ Unlike conventional lead-based perovskites, Ag–Bi systems are generally non-toxic and environmentally friendly, making them attractive for green energy applications.^152^ Their tunable electronic properties enable efficient charge separation and transport, crucial for enhancing photocatalytic activity. In PECs, Ag–Bi PIMs function as photoelectrodes, facilitating charge injection and reducing overpotentials. Surface engineering and heterostructure design further improve their performance by minimizing recombination losses. These materials can also exhibit synergy with co-catalysts, boosting reaction kinetics for different reactions. Understanding the role of defect chemistry and interfacial charge dynamics is key to optimizing their efficiency. With continued advancements, Ag–Bi perovskite-inspired photocatalysts hold great promise for next-generation solar-to-chemical energy conversion technologies.

**Fig. 8:**
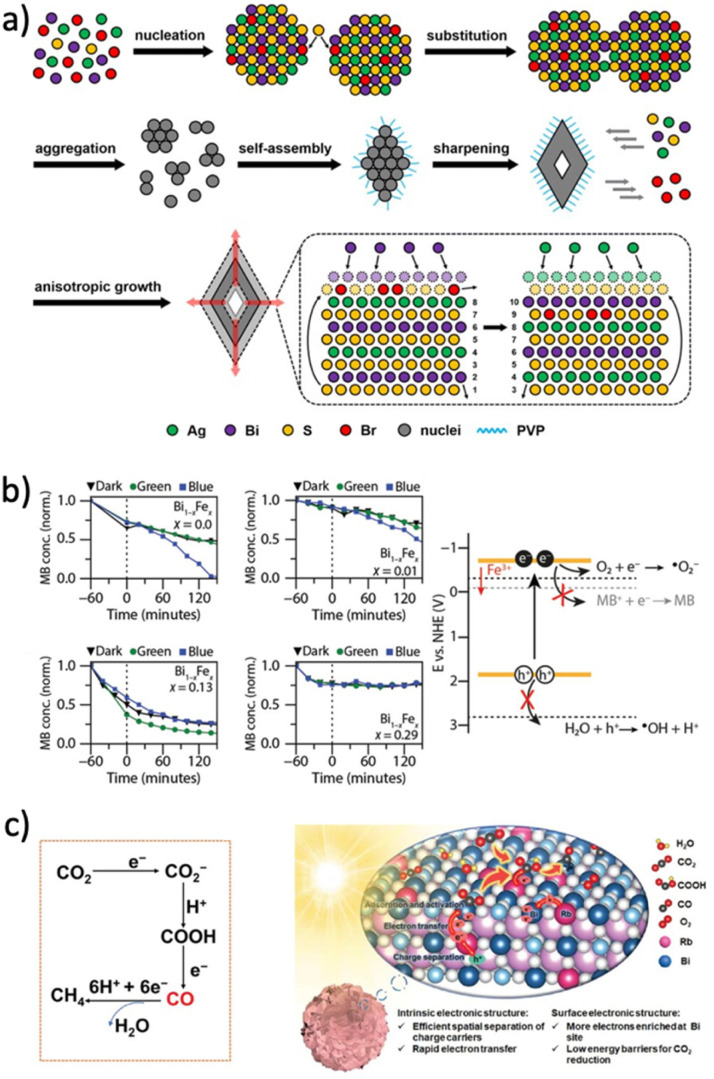
(a) Schematic illustration showing the growth mechanism of AgBiS_2_ nanocrystals through anion exchange. Reproduced from ref. 157 with permission from the Royal Society of Chemistry, Copyright 2024. (b) Methylene blue photocatalytic degradation by Fe-doped Cs_2_AgBiBr_6_ at different doping ratios and process mechanisms. Reproduced from ref. 158 with permission from Wiley-VCH, copyright 2023. (c) Mechanism of photocatalytic CO_2_ reduction by Rb-doped Cs_2_AgBiBr_6_ and effect of surface atom substitution. Reproduced from ref. 159 with permission from Wiley-VCH, copyright 2024.

Recent research on Ag–Bi PIMs has explored various compositions, including halides (*e.g.*, AgBiI_4_ and Ag_3_BiI_6_) and chalcogenides (*e.g.*, AgBiS_2_ and AgBiSSe). These materials exhibit tuneable bandgaps, making them suitable for visible-light-driven PCs and PECs. Halide-based compounds demonstrate strong light absorption and efficient charge transport,^153–155^ while sulfides offer improved stability and catalytic activity in aqueous environments.^156^ Hybrid systems, such as Ag–Bi chalcogenides combined with co-catalysts or heterostructures, are also gaining attention for enhanced performance in hydrogen evolution and CO_2_ reduction reactions. Zhang and co-workers utilized AgBiS_2_ as a catalyst for light-driven CO_2_ reduction, using zeolitic imidazolate frameworks as the co-catalyst.^157^ In this work, particular attention has been paid to engineering the growth mechanism of the nanocrystals, by inducing control of the rate of crystal growth and yielding a highly ordered structure by stabilizing the (111) facets. This was achieved by inducing atomic defects through partial Br^−^ ion doping during the nucleation phase and hence slowing down the crystal growth, which occurred through anion exchange with S^2−^ (Fig. 8a). This process, coupled with the capping effect of poly(vinyl pyrrolidone), yielded hollow octahedral particles with good light harvesting abilities and electron transfer capability, and hence is a very promising photoactive material with high potential as a support for zeolitic imidazolate frameworks (ZIF) co-catalysts. Moreover, AgBiS_2_ has demonstrated remarkable resistance to prolonged water exposure,^160^ making it a strong candidate for solar-to-hydrogen conversion *via* water splitting. Choi and co-workers were the first to report a PEC water-splitting system with AgBiS_2_ photoanodes, which featured broad light absorption and photocurrent generation primarily in the visible and near-infrared range.^161^ Their study also highlighted the significant impact of ligand selection on the PEC performance, emphasizing the importance of surface chemistry in optimizing the device efficiency.

For AgBiS_2_, the effect of CD has been extensively studied in terms of bandgap engineering,^90^ showing that the bandgap can be significantly reduced transitioning from the hexagonal semiconducting phase to the cubic metallic phase, enabling potential modulations of the photo(electro) chemical ability of the material. However, despite being frequently mentioned, the specific influence of CD and intrinsic defects in Ag–Bi chalcogenides on PC and PEC systems remains largely unexplored, highlighting a clear need for further investigation.

Doping has emerged as a powerful strategy for enhancing the photocatalytic and photoelectrocatalytic properties of Ag–Bi PIMs by tuning their electronic structure, improving charge carrier dynamics, and increasing stability. Introducing dopants can modulate the bandgap, optimizing light absorption and extending activity into the visible spectrum.^162^ Additionally, doping can enhance charge separation by creating defect states or internal electric fields that facilitate carrier migration, thereby reducing recombination losses. Structural modifications induced by dopants can also improve crystallinity and introduce strain effects that benefit catalytic performance. Moreover, stability—a key challenge in PIMs—can be enhanced by doping strategies that mitigate ion migration, suppress phase degradation, and reinforce the perovskite lattice. Recent studies have explored the potential of doped Cs_2_AgBiBr_6_ double perovskites for photocatalytic applications, focusing on band structure modulation and charge carrier dynamics. Hutter and co-workers investigated Fe-doped Cs_2_AgBiBr_6_, demonstrating that Fe incorporation effectively tunes the conduction band edge, enhancing charge separation and extending light absorption into the visible range.^158^ Through controlled alloying, Fe doping introduces mid-gap states that facilitate electron transfer, ultimately improving the photocatalytic efficiency (Fig. 8b). Fe-doped Cs_2_AgBiBr_6_ also exhibits greater structural stability under operational conditions, as the incorporation of Fe strengthens the perovskite lattice and reduces halide volatilization. Their work provides further insights into the defect chemistry of Fe-doped Cs_2_AgBiBr_6_, showing that Fe inclusion alters the charge compensation mechanisms and affects the carrier mobility, which is crucial for optimizing photocatalytic performance. Similarly, Chen *et al.* examined Rb-doped Cs_2_AgBiBr_6_ hierarchical microspheres for CO_2_ reduction, showing that Rb-doping enhances crystallinity, carrier mobility, and structural stability.^159^ The improved morphology increases active sites for CO_2_ adsorption, leading to higher photocatalytic efficiency (Fig. 8c). Both studies address key challenges in Cs_2_AgBiBr_6_-based photocatalysis, including limited absorption and charge recombination, by leveraging dopant-induced electronic and structural modifications. Additionally, the dopants contribute to improved stability, mitigating common degradation pathways such as ion migration. These findings underscore the potential of doped Cs_2_AgBiBr_6_ as a lead-free alternative for sustainable photocatalysis. Future research should focus on optimizing dopant concentrations, exploring synergies with co-catalysts and assessing long-term durability under operational conditions.

Ramachandran *et al.* reported a novel synthesis of lead-free Ag_2_BiI_5_ thin films *via* iodization of Ag/BiI_3_ layers, demonstrating excellent visible-NIR light absorption (bandgap, 1.2–1.6 eV).^163^ The films exhibited remarkable photocatalytic activity, degrading 96% of methylene blue (MB) dye under visible light in 120 minutes, attributed to their high crystallinity and low micro-strain. Silver(i) can also be partially replaced by copper(i), to yield mixed species. Liu *et al.* reported the first colloidal synthesis of water-resistant Cu_1.4_Ag_0.6_BiI_5_ nanocrystals (NCs), achieving a record 2.94% applied bias photon-to-current efficiency (ABPE) for lead-free photoelectrodes in water splitting.^164^ The layered structure and hydrophobic ligands of the NCs confer exceptional stability in aqueous media, while their bandgap (2.19 eV) aligns well with redox potentials for oxygen evolution. A photocurrent density of 4.62 mA cm^−2^ at 1.23 V *vs.* RHE was achieved without protective layers, surpassing previous Pb-free benchmarks.

Integrating Ag–Bi PIMs with co-catalysts or functional species is a convenient approach not only to enhance the photocatalytic and photoelectrochemical efficiency but also to improve the stability.^162^ Furthermore, heterostructure engineering with other semiconductors improves charge separation and passivates defect sites, extending material durability under operational conditions. Ravelli and co-workers recently demonstrated the synergistic combination of Cs_2_AgBiCl_6_ with graphitic carbon nitride (g-C_3_N_4_), highlighting its impact on enhancing photocatalytic efficiency in a wide series of carbohalogenation reactions.^165^ The heterojunction structure ensures improved charge separation and stability, addressing the common limitations of single-component photocatalysts.

In summary, Ag–Bi PIMs offer great promise for PC and PEC applications, yet several challenges remain to be addressed to fully realize their potential. Key limitations include relatively low charge carrier mobility, incomplete understanding of the role of CD and intrinsic defects, and concerns regarding long-term stability under operational conditions, especially in aqueous media. Achieving scalable and reproducible synthesis, particularly for doped systems and nanostructured heterojunctions, requires further refinement. Moreover, optimizing dopant concentrations and surface ligands to precisely tailor electronic structures and enhance charge separation remains a critical task. While combining Ag–Bi PIMs with co-catalysts or forming heterostructures has shown encouraging results, the complex interfacial charge dynamics still need to be thoroughly understood. Future work should integrate advanced *in situ* characterization and computational modelling to elucidate defect behaviour and provide guidance for rational material design. By addressing these aspects, Ag–Bi PIMs could emerge as key players in the development of efficient, stable, and lead-free systems for solar-driven fuel production and environmental remediation.

### 4.3. Memristors

The rapid advancement of artificial intelligence and the Internet of Things is driving the demand for efficient solutions in in-memory computing, encryption, edge computing, healthcare, wearables,^166,167^ and hardware security, including technologies such as physical unclonable functions.^168^ At the core of these applications are memristors, which uniquely integrate data processing and high-density storage within a single device.^17^ Their capabilities in real-time decision-making, low-power operation, and efficient data handling make them essential components for next-generation intelligent systems. To meet these performance demands, the development of innovative materials is critical.^17,166,167^ Recently, PIMs, including Ag–Bi materials, have emerged as promising candidates for advanced memristive applications due to their unique combination of electronic, ionic, and optical properties, along with their structural and environmental stability.^17,169,170^ The resistive switching (RS) behaviour and reliability of memristors are strongly influenced by the physical and optoelectronic characteristics of the PIM-based RS layer.^171^ Key factors include film thickness, morphology (including surface quality, roughness, grain size, and grain boundary density), chemical composition, and the presence of intrinsic or process-induced defects.^171,172^ The formation and rupture of conductive filaments, central to RS behaviour, are closely linked to the migration of point defects,^17^ highlighting the critical role of crystal structure and structure–property relationships in determining memristor performance.^173,174^ This review emphasizes the correlation between compositional engineering, particularly the choice of A-site cations and X-site halides, and the crystal structure and dimensionality of PIM-based RS layers, offering insights into their impact on memristive device performance.

Compositional engineering, particularly the selection of A-site cations and X-site halides, plays a pivotal role in defining key device parameters,^169,174,175^ such as the RS window (on/off ratio and set/reset voltage), endurance, and retention.^174,176,177^ While 3D structures are advantageous for photovoltaic and photodetector applications, lower-dimensional structures (0D, 1D, and 2D) offer enhanced stability and performance in memristors.^177,178^ Among these, the A-site cation is particularly influential: larger cations can significantly enhance the on/off ratio (from 10^3^ to 10^8^) and improve both thermal and environmental stability.^175,177–179^ For instance, incorporating butylammonium (BA) into Cs_2_AgBiBr_6_ yields the PIM BA_2_CsAgBiBr_7_. This distorted 2D structure facilitates ion migration and enhances RS behaviour. Devices based on BA_2_CsAgBiBr_7_ exhibit high on/off ratios (>10^7^–10^8^), likely due to a higher Schottky barrier at the electrode interface and constrained charge transport from deep trap states.^175,177–179^ As discussed earlier, halide composition significantly influences the structural dimensionality of PIMs, thereby affecting their thermal stability and memristor performance. For example, Cs_2_AgBiBr_4_Cl with its high chlorine content, demonstrates markedly improved characteristics, including enhanced data retention, greater endurance, and stable operation at temperatures up to 100 °C. These advantages are primarily attributed to its high activation energy, strong cohesive forces, reduced halide vacancy migration, and lower free carrier concentration, all contributing to superior thermal stability.^180^ Similar trends in A-site and X-site compositional engineering and their correlation with structural dimensionality and RS performance have been observed in various PIMs, including Cu_*x*_AgBiI_4+*x*_,^169^ BA_4_AgBiBr_8_,^179^ and Cs_2_AgBiBr_6−*x*_Cl_*x*_.^180^ The RS mechanisms in these systems are primarily governed by either metallic filaments (in devices with electrochemically active electrodes) or anionic conductive filaments based on halide vacancies.^17^ However, the influence of CD and cation vacancies on memristor performance remains largely unexplored and warrants further investigation. While some studies have examined the role of the crystal phase in LHPs (*e.g.*, FAPbI_3_),^173^ further exploration of polymorphism in other PIMs,^181^ including Ag–Bi systems, is imperative. Additionally, polymorphism must be considered alongside device configuration, particularly electrode selection, as a high interfacial energy barrier, resulting from work function mismatch, is often desirable for optimizing performance. Collectively, these insights pave the way for a new paradigm for highly stable and robust all-inorganic perovskite-type structures, deepening our understanding of how structural dimensionality and composition influence memristor properties.

Conventional memristive materials for neuromorphic devices include transition metal oxides (*e.g.*, TiO_2_ and HfO_2_) and halide perovskites (*e.g.*, MAPbI_3_).^182,183^ Ag–Bi PIMs exhibit comparable resistive switching while offering the advantages of being lead-free and environmentally benign.^13,184,185^ Their defect-tolerant, mixed ionic-covalent structure makes them promising for low-power synaptic devices.^17,184^ While transition metal oxides provide excellent endurance, they require high-temperature processing,^183^ whereas Ag–Bi PIMs are solution-processable at low temperatures, enabling compatibility with flexible electronics. However, their long-term stability and scalability need further optimization.^13,184^ Recent studies also demonstrate that Ag–Bi PIMs can integrate optoelectronic and memory functionalities, drawing parallels with oxide- and halide-based systems and opening pathways for multifunctional neuromorphic hardware.^184,186,187^

In conclusion, Ag–Bi PIMs represent a compelling class of materials for next-generation memristors, bridging the gap between the reliability of oxides and the tunability of halide perovskites, while eliminating lead toxicity. Continued progress in compositional engineering, defect control, and interface optimization will be critical to achieving the endurance, retention, and scalability required for practical deployment in neuromorphic and in-memory computing architectures.

### 4.4. Nonlinear optics (NLO)

Given the significant potential of Ag–Bi PIMs for NLO applications, the incorporation of chiral organic cations capable of breaking the crystal centrosymmetry has been actively explored. Notably, the chiral cation *R*-/*S*-β-methylphenethylammonium (*R*-/*S*-MPA) was used to synthesize the chiral double perovskite (*R*-/*S*-MPA)_4_AgBiI_8_ that was employed in the form of single crystals within a planar-type self-powered circularly polarized light photodetector.^188^ The device operates *via* the bulk photovoltaic effect, enabling efficient generation and separation of photogenerated carriers. Remarkably, it demonstrates superior discrimination between left- and right-handed circularly polarized light compared to Pb-based analogues. This enhanced performance was attributed to spin-polarization induced by orbital angular momentum since the heavy Bi and I atoms give rise to substantial spin orbit coupling, leading to significant Rashba spin splitting in the material.^188^

In another study, single crystals of the 2D Dion-Jacobson double perovskite (*R*-/*S*-4APEA)_2_AgBiI_8_·0.5H_2_O (*R*-/*S*-4APEA = *R*-/*S*-(4-aminophenyl)ethylamine) were employed in a circularly polarized light photodetector. The material exhibited a low defect density, which is essential for achieving efficient charge transfer while simultaneously suppressing the dark current.^189^ As in previous cases, the non-centrosymmetric crystal structure enabled the generation of an internal electric field, allowing the device to operate under zero-bias conditions. Furthermore, the intrinsic properties of the material facilitated the activation of the pyro-phototronic effect, which produces an additional photocurrent in response to light-induced temperature changes. This synergistic mechanism led to a 40-fold enhancement in both responsivity and detection compared to devices relying solely on the bulk photovoltaic effect.^189^ Interestingly, although photon energies above 520 nm are insufficient to drive charge separation and transport due to the intrinsic band structure of the semiconductor, the pyro-phototronic effect remains active up to 940 nm, enabling the extension of the operational spectral range of the circularly polarized light photodetector into the NIR region.^189^

The previously discussed (*R*-/*S*-MPA)_4_AgBiI_8_ was also employed to build up a self-powered X-ray photodetector, demonstrating a notably flat and smooth morphology. These high-quality films—characterized by low defect density—are advantageous for efficient charge transport during direct X-ray detection. The material exhibited a short PL lifetime, an ideal feature for photodetectors, as it reflects strong exciton binding energy together with substantial bulk resistivity, both of which contribute to minimizing dark current and noise. Benefiting from the bulk photovoltaic effect, which enables operation at zero bias, the fabricated photodetectors achieved higher sensitivity than those based on achiral perovskites even when operated under high external bias. It must also be mentioned that the detection limit degrades upon increasing the bias due to amplification of dark current and noise induced by ion migration, highlighting the benefits of developing inherent non-centrosymmetric materials for self-powered devices.^190^

A dicationic chiral molecule, namely *R*-/*S*-/*Rac*-3-aminopyrrolidine (*R*-/*S*-/*Rac*-3AP), was used for the synthesis of (*R*-/*S*-/*Rac*-3AP)_4_AgBiBr_12_ in the form of both polycrystalline powders and highly crystalline microwire arrays oriented along the 001 crystallographic axis.^191^ Second harmonic generation (SHG) studies were performed, observing a higher SHG conversion efficiency for the microwire arrays upon equal excitation intensity, ascribed to grain boundary suppression, inferior propagation loss, and pure (001) orientation.^191^

Remarkably, in 2025, non-centrosymmetric structural centers were successfully introduced into the globally centrosymmetric structures of CABI and AgBiI_4_ without the use of chiral cations.^12^ In these materials, local centrosymmetry breaking was achieved through the presence of cation vacancies. Namely, while ions or atoms are symmetrically arranged with respect to an inversion center in centrosymmetric crystals without defects, cationic or atomic vacancies can locally lead to an unbalanced displacement of atoms disrupting the centrosymmetric nature of the crystal lattice. A stronger SHG signal was observed for CABI compared to AgBiI_4_, consistent with a higher concentration of cationic defects in the former. This finding highlights defect engineering as a powerful strategy for inducing NLO responses in metal halide systems without the need for chiral cations.^12^ This approach is particularly appealing, given the intrinsic steric limitations of chiral cations, which make the attainment of 3D structures difficult and restrict efficient charge transport along all crystallographic directions.^192^ Moreover, the thermal instability of organic molecules significantly restricts the application range of hybrid materials.

Although research interest in Ag–Bi PIMs has grown significantly in recent years, the number of comprehensive studies on NLO properties remains limited. There is an urgent need for a more systematic approach to assess how key parameters—including chemical composition, octahedral distortion, cation vacancies, network dimensionality, and morphology—affect their NLO properties. The incorporation of chiral organic molecules ensures global non-centrosymmetry across the material, thereby enabling second-order NLO responses such as SHG. In contrast, fully inorganic compounds like CABI and AgBiI_4_ exhibit SHG signals that predominantly originate from localized surface regions, where cation vacancy concentrations are higher. These materials, however, benefit from a fully three-dimensional connectivity of the inorganic framework, without the insulating organic spacer layers typically present in hybrid structures. In this context, the realization of a chiral 3D Ag–Bi PIM—analogous to recent developments in Pb-based systems—represents a highly promising direction.^193^

Comprehensive studies are essential to elucidate how the electronic and steric characteristics of chiral cations, along with the halide composition, influence the resulting optoelectronic and NLO properties. Parallel efforts toward the development of fully inorganic Ag–Bi PIMs should focus on expanding the family of compounds studied, integrating both experimental and theoretical approaches. This includes quantifying the role of octahedral distortion indices and cation vacancy distributions, as well as engineering defect structures and optimizing film-processing conditions, with the ultimate goal of maximizing the NLO functional response.

### 4.5. Emerging energy storage, sensing, and radiation detection applications

Beyond photovoltaics and optoelectronic applications, Ag–Bi PIMs have recently emerged as promising candidates for energy storage, sensing, and radiation detection.

AgBiS_2_, for instance, has demonstrated notable potential as an anode material in alkali-ion batteries. Its nanocrystals exhibit reversible capacities (∼420 mAh g^−1^) and remarkable cycling stability in potassium-ion battery systems, driven by reversible Ag/Bi redox reactions and efficient ion diffusion pathways.^194^ In addition, AgBiS_2_ has also been explored for charge-storage applications, achieving a specific capacitance of approximately 14 F g^−1^ at 2 mV s^−1^, an energy density of 26 Wh kg^−1^, and a power density of 3.6 kW kg^−1^.^195^ The inherent CD and mixed ionic-electronic conductivity significantly enhance its energy-storage capabilities, underscoring its potential for high-performance supercapacitors. Additionally, AgBiS_2_ demonstrates efficient near-infrared-driven photocurrents (∼2.3 mA cm^−2^ at ∼800 nm), sufficient to enable neuronal stimulation, thus suggesting its potential use in bioelectronic interfaces, such as neural implants and biosensors.^196^

Furthermore, Ag–Bi halide materials, particularly Cs_2_AgBiBr_6_, have emerged as promising candidates for solid-state X-ray detection. Their heavy-atom lattice structure combined with defect-tolerant wide bandgaps results in exceptional hard X-ray sensitivity (∼1390 µC Gy^−1^ cm^−2^ at 100 keV), ultralow dark current drift (1.03 × 10^−8^ nA cm^−1^ s^−1^ V^−1^), and remarkably low detection limits (<100 nGy_air_ s^−1^), outperforming commercial CZT detectors in certain key metrics.^197^ These properties position Cs_2_AgBiBr_6_ and related Ag–Bi PIM-based detectors as competitive alternatives to conventional inorganic X-ray detectors, offering distinct advantages such as improved stability, lower toxicity, and enhanced environmental friendliness. Interestingly, due to their typically short charge-carrier diffusion lengths arising from strong coupling with lattice vibrations, Ag–Bi halide PIMs are particularly well-suited for drift-driven applications such as radiation detection, rather than diffusion-driven photovoltaic devices.^14^ Under applied electric fields, these materials can achieve notably high drift mobility-lifetime (*µ*_*τ*_) products, making them promising candidates for high-performance radiation detectors. Notably, recent studies have highlighted Cs_2_AgBi_2_I_9_ for achieving the highest reported *µ*_*τ*_ product (3.4 × 10^−3^ cm^2^ V^−1^) among Bi-halide PIMs.^41^

Collectively, these emerging energy storage, sensing, and radiation detection applications highlight the multifunctional potential of Ag–Bi PIMs, suggesting significant opportunities beyond traditional photovoltaic or optoelectronic domains.

### 4.6. Scalability, reliability, and adoption

Across the Ag–Bi PIM families, credible routes now exist to scale thin-film growth beyond spin coating while preserving device-grade quality. For Cs_2_AgBiBr_6_, dry/semidry methods—including sequential vapor deposition, pulsed layer deposition, chemical vapor deposition, ultrasonic spray, and blade coating—have produced compact, uniform films compatible with patterning, crossbar arrays, and pixelated devices.^133,142,198–200^ Performance bands reported for these routes (*e.g.*, sequential vapor ≈1.5% 1-sun PCE, spray ≈2.3% 1-sun PCE, a high solar cell *V*_OC_ of 1.09 V, a fast (rise/fall) photoresponse of ∼170/177 µs, and 6 mW power output from a 6 cm^2^ solar module) provide practical context for replication. AgBiS_2_ has progressed on parallel tracks: phase-pure thermally co-evaporated films and vapor-assisted conversion/chemical vapor deposition deliver dense absorbers (small-area, 10.20%; 1.00 cm^2^, 9.53%) with strong durability (*e.g.*, ≥94% PCE after 3000 h ambient; 87% after 1000 h at 85 °C), while NC inks enable benign, printing-compatible processing—a practical bridge to roll-to-roll once ink rheology and drying kinetics are tuned.^11,201,202^

CABI and Cs_2_AgBi_2_I_9_ remain predominantly solution-processed (co-evaporated CABI films exist but are transport-limited),^33^ yet reproducible planar/mesoscopic device stacks—including indoor-PV architectures—are now routine. By contrast, chalcohalides (*e.g.*, AgBiSCl_2_) are successfully synthesized at the nanocrystal level (direct bandgap, robust films)^10^ yet still lack module-relevant, large-area thin-film processes—an obvious near-term target for process development.

Long-term photovoltaic stability in Ag–Bi semiconductors is increasingly supported by targeted chemistry and interfaces. In Cs_2_AgBiBr_6_, hydrogenation narrows the bandgap (≈2.18 → ≈1.64 eV) and delivers steady solar cell performance under illumination at 85 °C with a record PCE of 6.37%, indicative of drift-suppressed behaviour under coupled heat/light stress. Mechanistically, interstitial H tunes band edges and reduces halide-vacancy-related traps.^134^ Beyond hydrogenation, interface/dopant engineering has delivered ≈90% PCE retention after ambient and heat aging (unencapsulated) in Cs_2_AgBiBr_6_—consistent with a picture where contact chemistry and defect passivation dominate operational loss channels.^203^ Light-stress studies further confirm that while the bulk lattice is thermally rugged (to ∼410 °C), photo-driven pathways can emerge at higher intensities or with poor interfaces, underscoring the importance of ion-migration barriers and surface passivation in stack design.^204^

In CABI, non-encapsulated solar cells retained most of their performance for a few weeks, and halide/cation/interface engineering further suppresses non-radiative losses and enhances device stability.^43,45,135^ AgBiS_2_ thin-film devices—when densified by solution crystallization or vapor-assisted routes—exhibit continuous 1-sun stability and months-long dark storage, mitigating the durability limits of ligand-rich NC-only layers. Vapor-assisted, solution-processed AgBiS_2_ solar cells have also exhibited excellent stability—from hundreds up to ∼3000 h under storage in ambient humidity and 85 °C.^11,137^

#### 4.6.1 Operational stability—ISOS (international summit on organic solar cells stability) protocols/international electrotechnical commission (IEC) framing

To make Ag–Bi PIM stability comparable across labs, outdoor testing with ISOS protocols (stabilized MPP, *T*_80_; defined light/heat/humidity stresses)^205^ and indoor testing with IEC TS 62607-7-2:2023 (fixed illuminance, spectrum reporting, and stabilized output at 50/200/1000 lux)^206^ are necessary. Recent Ag–Bi studies now report indoor PCE with lux-specified WLED spectra, allowing reproducible comparisons. For Cs_2_AgBiBr_6_, hydrogenation not only raises efficiency but also demonstrates ISOS-style thermal/light robustness.^134^ Comprehensively, ISOS-style operational datasets remain sparse across some Ag–Bi halide PIMs. Interface chemistry determines both the long-term storage and operational stability of CABI solar cells, further emphasizing that interfacial chemistry has a major role in improving the device stability of Ag–Bi halide PIMs.^42^

For AgBiS_2_, quantitative ISOS-type datasets show ≥94% PCE retention after ∼3000 h ambient and ∼87% after 1000 h at 85 °C, while shorter operational MPP traces highlight the importance of publishing both shelf and operational stability.^11^ State-of-the-art NC-based AgBiS_2_ solar cells demonstrated *T*_80_ of just ≈15 h (≈900 min) under maximum power point tracking.^207^ This is significantly lower operational stability compared to LHPs, which routinely demonstrate thousands of hours of operational stability. These examples collectively illustrate that adopting ISOS/IEC practices is essential for cross-lab comparability and benchmarking Ag–Bi PIMs against LHPs and other emerging/established PV technologies. For indoor PV, the operational stability data are very limited; Cs_2_AgBi_2_I_9_ maintains *T*_80_ ≈ 35 h under continuous 1000 lux illumination with MPP tracking.^208^

#### 4.6.2 Degradation pathways (outdoor and indoor PV)

Under outdoor (1-sun, heat/humidity) stress, the best-resolved mechanisms in Cs_2_AgBiBr_6_ involve ionic motion and interfacial reactivity: Ag^+^/Br^−^ diffusion into the HTL drives long-term decay; Ag electrodes can form AgI/AgBr at the interface.^209^ These trends align with the hydrogenation-enabled operation at 85 °C noted above and with reports of faster degradation in early unpassivated films under air + light.^30^ At the interface level, BiOBr heteroepitaxy blocks halide/ion motion and stabilizes electrodes.^117^ For AgBiS_2_, surface/ligand-mediated channels dominate in NC stacks (oxygen/light reactions and contact-induced recombination), whereas densified films sustain steady 1-sun operation and long storage—*i.e.*, microstructure compaction largely eliminates NC-specific routes.^137^ Under indoor (50/200/1000 lux) conditions, thermal/humidity stresses are milder and interfacial electronic drift and transport-layer aging dominate; the field increasingly employs IEC TS 62607-7-2:2023, improving cross-lab comparability of failure modes.

For memristors, stability manifests as low-voltage switching with quantifiable endurance and retention. Cs_2_AgBiBr_6_ devices operating near +0.4/−0.3 V with on/off ≈4.8 × 10^2^, ≈10^3^ cycles, and ≈10^4^ s retention exemplify the current window; failure modes map to ionic/filamentary drift and electrode reactivity, which are mitigated by interlayers and film densification.^184^ AgBiS_2_ quantum-dot memristors demonstrate multilevel switching at very low set power (∼0.065 mW) and ≈500-cycle pulsed endurance, consistent with Ag-ion migration and *S*-vacancy-assisted filaments. This is again pointing to barrier/electrode chemistry as the principal means for drift suppression.^210^ In photocatalysis/PEC, Ag–Bi materials show encouraging *operando* resilience when interfaces are engineered. AgBiS_2_ solids are water-resistant at the film level (pre-/post-XPS/XRD show no compositional/structural change after water immersion), and neutral-electrolyte PEC operation is documented.^160,161^ Epitaxial ZnS shells suppress surface traps/corrosion, which can extend the steady-state operation.^47^ Long-run device-level datasets are currently unavailable for chalcohalides (*e.g.*, AgBiSCl_2_).

To improve reproducibility, we align explicitly with ISOS (outdoor; stabilized MPP, *T*_80_, and defined light/heat/humidity) and IEC TS 62607-7-2:2023 (indoor; 50/200/1000 lux, spectral reporting, and stabilized output). Because interfaces and defect chemistry repeatedly control stability across applications, a pragmatic round-robin on one halide (Cs_2_AgBiBr_6_) and one chalcogenide (AgBiS_2_)—covering ISOS/IEC PV, memristor endurance/retention, and PEC steady-state with FE/ICP-MS—would separate materials from tool/process effects and accelerate the consensus on lifetimes and efficiency levers.

#### 4.6.3 Commercialization barriers and market readiness

Regarding scalability and cost, Ag–Bi PIMs benefit from low-temperature solution printing (inks and slot-die/blade) and dry vacuum routes (sequential vapor, single-source evaporation, pulsed layer deposition, and chemical vapor deposition), all compatible with high-throughput manufacturing. Recent techno-economic analyses of perovskite roll-to-roll lines indicate the low film cost at scale (≈$0.10 W^−1^),^211^ suggesting that similar CAPEX/OPEX advantages are plausible for PIMs once efficiencies and yields mature. Supply-chain factors are non-trivial: silver is widely used (electronics and investment) with price/availability volatility, and bismuth supply is concentrated geographically and often by-product-limited^212,213^—considerations for cost and sourcing strategies in scaled products. Regarding market readiness, the nearest applications are IPV—where performance and stability are promising under IEC-aligned tests^8^—and direct X-ray detection, where Cs_2_AgBiBr_6_ single-crystal detectors show a low drift and limit of detection of 59.7 nGy s^−1^.^214^ For neuromorphic devices, demonstrations of low-voltage, multilevel operation position Ag–Bi PIMs for edge/neuromorphic niches as endurance/retention metrics are standardized.

## 5. Conclusions and outlook

Ag–Bi PIMs have demonstrated their potential as lead-free photovoltaic absorbers and as a versatile platform for multifunctional integration in electronics and photonics. Ag–Bi PIMs are environmentally benign materials that, when combined with unique structural features (such as disorder, vacancies, and heavy-element chemistry), offer exciting opportunities for next-generation devices. Recent studies already highlight this potential—for example, Ag–Bi PIMs exhibited strong SHG for on-chip frequency conversion, demonstrated resistive switching suitable for neuromorphic memory devices, and functioned as highly sensitive X-ray detectors.

Under 1-sun (AM 1.5G), state-of-the-art LHPs reach ≈26–26.7% single-junction PCE, with hundreds of hours of operational stability already demonstrated in well-engineered stacks. By contrast, Ag–Bi PIM solar cells are still efficiency-limited. The best Ag–Bi halide PIM to date is hydrogenated Cs_2_AgBiBr_6_ at ∼6.4% (*J*–*V*) after narrowing the bandgap to ∼1.64 eV.^134^ Performance of Ag–Bi iodides remains lower outdoors (*e.g.*, Ag–Bi–I ≈ 4.5%, Cs_2_AgBi_2_I_9_ ≈ 3% and CABI ≈ 2.2% in recent optimized stacks),^7,8,136^ consistent with wide-gap/defect-limited *V*_OC_ and transport losses that remain to be solved. Ag–Bi chalcogenides now deliver ≥10% PCE (AM 1.5G), establishing the highest outdoor efficiency within Ag–Bi PIMs but still less than half of the LHP's record value.^11,207^ Under indoor lighting (WLED), the comparison is more nuanced because the optimal bandgap shifts to ≈1.9 eV. Ag–Bi iodides sit near this optimum bandgap and avoid the mixed-halide phase-segregation commonly encountered when LHPs are tuned to 1.9 eV for indoor use. Theoretical, radiatively limited indoor maximum efficiency (i-SLME) ceilings for many Ag–Bi iodides approach 50%.^140^ Experimentally, Ag–Bi PIMs have progressed from early 4–5% IPV (Ag–Bi–I) to ≈8% at 1000 lux for Cs_2_AgBi_2_I_9_.^8^ These values remain below best-in-class LHP IPV reports (>40%) but demonstrate that the Ag–Bi family is competitive in bandgap suitability and increasingly credible in device metrics under standardized indoor testing.

Although Ag–Bi PIMs significantly trail state-of-the-art LHPs for photovoltaics, additive/interface engineering continues to improve film quality and device metrics—material levers that are equally consequential for radiation detectors and memristors.

Benchmarks for Cs_2_AgBiBr_6_ include sensitivities up to ≈1974 µC Gy^−1^ cm^−2^ with appropriate interface passivation and limits-of-detection around 59.7 nGy s^−1^.^214^ Polycrystalline thick-film Cs_2_AgBiBr_6_ has also shown a sensitivity of ≈487 µC Gy^−1^ cm^−2^, and related Ag–Bi compounds (*e.g.*, AgBi_2_I_7_) report limits-of-detection of ≈72 nGy s^−1^.^215^ For context, legacy a-Se panels offer ≈20 µC Gy^−1^ cm^−2^ sensitivity, while LHP single-crystal detectors span ≈9300–24,552 µC Gy^−1^ cm^−2^ sensitivity with limits-of-detection ≈0.22–54 nGy s^−1^ depending on the architecture and crystal quality.^216,217^ Cs_2_AgBiBr_6_ has also shown 59.5 keV γ-ray response with ≈13.9% energy resolution and measurable responses at 511 and 662 keV (ref. 218)—evidence that Ag–Bi halide double perovskites can extend beyond diagnostic X-rays into higher-energy regimes. Thus, Ag–Bi detector benchmarks already clear the thresholds used to judge a-Se flat-panel photoconductors and close the gap to the best LHP single-crystal photodiodes, while offering an intrinsically Pb-free route and device-relevant stability when contact chemistry and ion migration are controlled.

In nonlinear optics/chiroptical detection, chiral Ag–Bi double-perovskite microwire arrays ((*R*/*S*-β-MPA)_4_AgBiI_8_) yield circular-polarization photodetection with *g*-factor ≈0.19, responsivity >52 mA W^−1^, and detectivity >3.9 × 10^11^ Jones—values competitive for device-level circularly polarized light sensing.^219^ Representative Pb-based chiral perovskite comparators can reach higher *g* (≈0.2) and responsivity near ≈0.8 A W^−1^,^220,221^ underscoring the headroom for Ag–Bi optimization while already enabling useful circularly polarized light discrimination in lead-free systems. Recent work by Fan *et al.* demonstrates that lead-free Ag–Bi-based double perovskites can deliver superior SHG performance compared to LHPs, primarily due to the highly distorted [AgBr_6_]^5−^ octahedra.^222^ These distortions break centrosymmetry more effectively than the relatively ideal [PbX_6_]^4−^ configurations in lead-based systems, enabling stronger nonlinear optical responses. The compound DFPD_4_AgBiBr_8_·H_2_O exhibits a record SHG intensity of 13 × KH_2_PO_4_ (KDP), significantly surpassing the best-performing lead-based analogs while also offering enhanced environmental stability and a high laser-induced damage threshold.^222^ This study, alongside the recent observation of SHG from non-centrosymmetric Cu–(Ag)–Bi–I thin films,^12^ emphasizes the unique promise of lead-free Ag–Bi PIMs for nonlinear optical applications.

In memristive switching, Ag–Bi PIMs demonstrate low-voltage operation and robust figures-of-merit: AgBiI_4_ devices switch at ≈0.16 V with on/off ≈10^4^, >700-cycle endurance, ≳10^4^ s retention, and stability under 1000 bending cycles;^185^ 2D Ag–Bi systems such as (BA)_2_CsAgBiBr_7_ report on/off >10^7^–10^8^ with improved retention.^175^ LHP memristors set the current performance ceiling—for example, quasi-2D devices with on/off ≈10^9^ (±0.8 V), ≈230 cycles and ∼10^3^ s retention, with broader reviews placing typical LHP on/off in the 10^3^–10^6^ range and endurance up to 10^3^–10^4^ cycles.^223,224^ These side-by-side values show Ag–Bi devices already competitive for low-voltage switching, with clear headroom in endurance scaling.

These diverse capabilities underscore that Ag–Bi PIMs are far more than niche photovoltaic absorbers—they are versatile functional semiconductors poised to meet the emerging needs in energy, computing, and sensing—with the important benefit of eliminating lead.

### 5.1. Key challenges and strategic focus

To fully realize these opportunities, several unsolved challenges must be addressed through coordinated research efforts. A key short-term priority is materials tuning—overcoming intrinsic limitations in Ag–Bi crystal and electronic structures (such as indirect bandgaps, low mobility, and CD), which currently hinder performance. Targeted compositional engineering and defect passivation will be essential. Alloying strategies that introduce subtle distortions or elemental substitutions may help increase bandgap directness and enhance carrier mobility, while advanced synthesis and processing techniques can suppress deep trap formation. High-throughput computational design will be invaluable at this stage. First-principles calculations and machine-learning models can effectively screen new Ag–Bi compositions or mixed-anion phases with improved electronic connectivity, defect tolerance, and operational stability. By focusing on atomic-level optimizations in the coming years, the research community can establish rational design rules to consistently produce Ag–Bi PIM films and crystals with high optoelectronic quality.

### 5.2. Roadmap for Ag–Bi PIM advancement

A conceptual development pathway for Ag–Bi PIMs can be outlined in three stages, aligning materials progress with device innovation.

#### 5.2.1 Material tuning

Optimizing chemistry and microstructure remains the immediate priority. Recent advances, such as the AI-driven Daisy framework for microstructural optimization in Ag–Bi–I materials like AgBiI_4_,^139^ demonstrate how data-guided synthesis can rapidly enhance grain quality and defect control. In parallel, the discovery of new Ag–Bi variants—such as alloyed halide-chalcogenides or A-site cation engineering (*e.g.*, Cs_3_Bi_2_I_9_ → (Cs_2_Ag)Bi_2_I_9_)—offers promising routes to tuneable bandgaps and improved EDs. Precise control over antisite disorder through stoichiometric tuning and effective passivation of vacancy defects are also critical. Success in these areas will be measured by improvements in carrier mobilities, lifetimes, synthesis reproducibility, and other tuneable optoelectronic properties. Collectively, these advances lay the foundation for high-performance, lead-free semiconductor devices.

#### 5.2.2 Device engineering

As a medium-term plan, the focus shifts to leveraging improved materials in prototype devices tailored for specific emerging applications. In photovoltaics, this entails designing IPV cells that consistently achieve >20% PCE under low lighting, enabled by the wide-bandgap Ag–Bi halides. Simultaneously, efforts will target the integration of Ag–Bi PIMs into photonic circuits (for frequency conversion and optical switching) and neuromorphic hardware (such as memristor crossbar arrays). Here, device physics and engineering become critical: optimizing charge transport in thin-film architectures, minimizing interfacial losses (*e.g.*, through a thin passivation layer), and developing scalable processing techniques compatible with CMOS and flexible substrates. Notably, Ag–Bi PIM memristors have already demonstrated excellent endurance and bending stability in flexible formats, indicating potential for wearable neuromorphic systems. In the medium term, we anticipate demonstrations of Ag–Bi PIM devices—from lead-free photonic modulators to self-powered sensor nodes—that either outperform or uniquely complement existing technologies.

#### 5.2.3 System-level integration

This phase focuses on the holistic integration of Ag–Bi PIM-based components into complex, multifunctional systems. At this stage, the vision becomes truly interdisciplinary. Potential applications include smart implants and bioelectronic systems—for example, a stable, optimized Ag–Bi PIM photovoltaic device that harvests ambient light to power embedded sensors or drug-delivery chips. Another promising direction is photonic–electronic hybrid processors, where Ag–Bi PIM optical elements (providing on-chip light generation or frequency doubling) interface directly with Ag–Bi memristive networks to enable brain-inspired optical computing modules. Realizing such ambitious systems will demand coordinated efforts involving materials chemistry (to ensure long-term, biocompatibility, and sustainability), advanced fabrication (to pattern and/or integrate multi-component circuits), device physics (to manage the interplay of ionic and electronic effects), and biomedical engineering (to tailor devices for human-proximity applications). Achieving full system-level integration is a long-term goal, but steady progress in materials development and device engineering could make it attainable.

Reaching these milestones will require a deeply interdisciplinary approach. Chemists, materials scientists, theoreticians, and device engineers must work to synchronize their approaches across domains—for example, by combining computational predictions with *in situ* spectroscopy and advanced imaging techniques to identify and eliminate performance-limiting defects. Collaboration with application-domain experts is equally important: as the demands for IPVs, photonic computing, and radiation sensing evolve, feedback from those communities will help steer Ag–Bi PIM development toward the most impactful and relevant solutions. Notably, the lead-free, low-toxicity nature of these materials is expected to reduce barriers to deployment in sensitive environments (from living spaces to clinical settings)—aligning with the global push for sustainable and safe electronics.

In summary, Ag–Bi PIMs are at a promising turning point. Having overcome key challenges related to stability and synthesis, the field is now poised to move beyond just catching up with LHPs and begin exploring new directions that those toxic materials could not safely reach. By systematically improving material quality in the short term, innovating device architectures in the medium term, and embracing system-level design in the long term, Ag–Bi PIMs have the potential to enable a new generation of clean energy harvesters, intelligent sensors, and hybrid optoelectronic–photonic systems.

The outlook is ambitious, but with creative research and sustained cross-disciplinary collaboration, Ag–Bi PIMs can evolve from a promising alternative into a transformative technology for next-generation energy, sensing, and computing platforms.

## Author contributions

G. K. G. and P. V. wrote the Abstract and Section 1 (Introduction). N. S. M. V. wrote Section 2 and 2.1 (Structural aspects and stability of Ag-Bi PIMs) and Section 3.5 and 3.6 (Defect chemistry and defect mitigation strategies of Ag-Bi PIMs). A. P., A. B. M.-G., and M. P. wrote Section 3.1 (Electronic band structure). M. R. wrote Sections 3.2 (Electronic dimensionality), 3.3 (Charge-carrier transport and optoelectronic properties), and 3.4 (Cation disorder and cation vacancies). N. S. M. V. and G. K. G. wrote Section 3.7 (Photophysical properties). G. K. G. wrote Sections 3.8 (Thin‑film deposition: recent progress and persistent challenges), 4.1 (Photovoltaics), 4.5 (Emerging energy storage, sensing, and radiation detection applications), and 4.6 (Scalability, eeliability, and adoption**)**. S. D. and T. G. wrote Section 4.2 (Photocatalysis and photoelectrocatalysis). M. K. wrote Section 4.3 (Memristors). M. M. and L. M. wrote Section 4.4 (Nonlinear optics). G. K. G. and P. V. wrote Section 5 (Conclusions and outlook). All authors contributed to editing the review. P. V. supervised the overall project.

## Conflicts of interest

There are no conflicts to declare.

## Data Availability

No new experimental or theoretical data were generated as part of this review.
